# Brain-behavior analysis of transcranial direct current stimulation effects on a complex surgical motor task

**DOI:** 10.3389/fnrgo.2023.1135729

**Published:** 2024-01-09

**Authors:** Pushpinder Walia, Yaoyu Fu, Jack Norfleet, Steven D. Schwaitzberg, Xavier Intes, Suvranu De, Lora Cavuoto, Anirban Dutta

**Affiliations:** ^1^Department of Biomedical Engineering, University at Buffalo, Buffalo, NY, United States; ^2^Department of Industrial and Systems Engineering, University at Buffalo, Buffalo, NY, United States; ^3^U.S. Army Futures Command, Combat Capabilities Development Command Soldier Center STTC, Orlando, FL, United States; ^4^University at Buffalo School of Medicine and Biomedical Sciences, Buffalo, NY, United States; ^5^Center for Modeling, Simulation, and Imaging in Medicine, Rensselaer Polytechnic Institute, Troy, NY, United States; ^6^Department of Biomedical Engineering, Rensselaer Polytechnic Institute, Troy, NY, United States

**Keywords:** transcranial direct current stimulation, surgical training, error based learning, fundamentals of laparoscopic surgery, electroencephalogram, functional near-infrared spectroscopy, cerebellar tDCS, ventrolateral prefrontal cortex tDCS

## Abstract

Transcranial Direct Current Stimulation (tDCS) has demonstrated its potential in enhancing surgical training and performance compared to sham tDCS. However, optimizing its efficacy requires the selection of appropriate brain targets informed by neuroimaging and mechanistic understanding. Previous studies have established the feasibility of using portable brain imaging, combining functional near-infrared spectroscopy (fNIRS) with tDCS during Fundamentals of Laparoscopic Surgery (FLS) tasks. This allows concurrent monitoring of cortical activations. Building on these foundations, our study aimed to explore the multi-modal imaging of the brain response using fNIRS and electroencephalogram (EEG) to tDCS targeting the right cerebellar (CER) and left ventrolateral prefrontal cortex (PFC) during a challenging FLS suturing with intracorporeal knot tying task. Involving twelve novices with a medical/premedical background (age: 22–28 years, two males, 10 females with one female with left-hand dominance), our investigation sought mechanistic insights into tDCS effects on brain areas related to error-based learning, a fundamental skill acquisition mechanism. The results revealed that right CER tDCS applied to the posterior lobe elicited a statistically significant (*q* < 0.05) brain response in bilateral prefrontal areas at the onset of the FLS task, surpassing the response seen with sham tDCS. Additionally, right CER tDCS led to a significant (*p* < 0.05) improvement in FLS scores compared to sham tDCS. Conversely, the left PFC tDCS did not yield a statistically significant brain response or improvement in FLS performance. In conclusion, right CER tDCS demonstrated the activation of bilateral prefrontal brain areas, providing valuable mechanistic insights into the effects of CER tDCS on FLS peformance. These insights motivate future investigations into the effects of CER tDCS on error-related perception-action coupling through directed functional connectivity studies.

## 1 Introduction

Transcranial direct current stimulation (tDCS) has demonstrated its efficacy in enhancing the acquisition and mastery of surgical skills when administered to specific cortical regions, such as the primary motor cortex (Cox et al., 2020; Gao et al., 2021a) and the prefrontal cortex (Ashcroft et al., 2020). A comprehensive meta-analysis of randomized control studies revealed that tDCS led to significantly superior performance in surgical tasks compared to sham control conditions (Hung et al., 2021; Walia et al., 2021). In a study on bimanual pattern cutting involving tDCS of the primary motor cortex (M1) and portable neuroimaging (Gao et al., 2021a), Gao et al. (2021b) observed a significant effect of M1 tDCS on performance errors (*p* < 0.001; *t-*test for normal distribution or Mann–Whitney *U*-test for non-normal distribution) after day 7 compared to the sham group. Notably, a delayed effect of M1 tDCS emerged after day 7, postulated to be linked to the significant activation of M1 observed only during the later learning stage (day 7–12) in contrast to the initial learning stage (day 2–6). This aligns with established *in vivo* effects of tDCS, which do not alter the firing rates of cortical neurons (Krause et al., 2017) but instead modulate endogenous task-specific brain activity (Woods et al., 2019). Therefore, neuroimaging can offer insight into the cortical “target” associated with endogenous task-specific brain activity (Walia et al., 2021).

**Figure 1:**
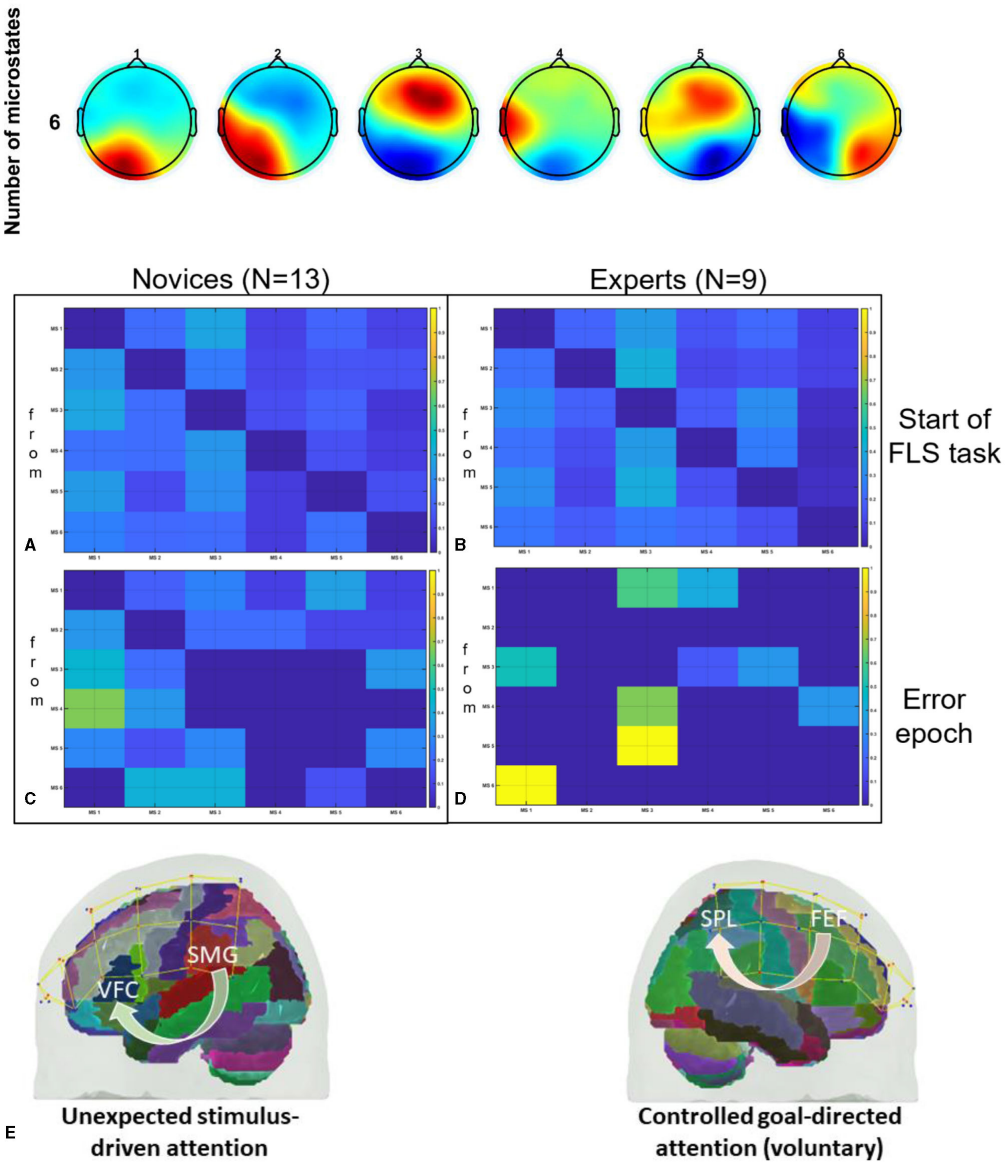
Top panel shows the six microstate prototypes discerned from the amalgamated EEG data encompassing both experts and novices performing the FLS complex task, elucidating 77.14% of the overall variance (Walia et al., 2022a). Then, the transition probabilities between microstate (MS) classes at the group level are shown, **(A)** during the 10 s at the start of the FLS laparoscopic suturing with intracorporeal knot tying task in novices, **(B)** during the 10 s at the start of the FLS laparoscopic suturing with intracorporeal knot tying task in experts, **(C)** during the 10 s in the error epoch in novices, **(D)** during the 10 s in the error epoch in experts. In the transition probability matrix, the rows denote the “from” microstate and the columns denote the “to” microstate. **(E)** A differentiation is proposed between attention driven by unexpected stimuli (errors) and controlled, goal-directed attention (voluntary) within the frontoparietal network system (Walia et al., 2022a). Here, ventrolateral prefrontal cortex (VFC), superior parietal lobule (SPL), supramarginal gyrus (SMG), frontal eye field (FEF), brain regions were considered. Figures adapted from Walia et al. (2022a).

Given that complex motor activities engage diverse brain regions associated with motor control and attention (Alahmadi et al., 2016), a thorough mechanistic understanding of the task-specific brain activity for region-specific tDCS targeting is necessary (Walia et al., 2021). For instance, the dorsolateral prefrontal cortex (PFC) plays a key role in goal-directed behavior and learning, involving coordinated activity within a broader frontoparietal network (James et al., 2013). Changes associated with visuomotor learning may encompass reduced activation in the PFC, coupled with increased activation in the primary and secondary motor regions, the cerebellum, and the posterior parietal cortex (James et al., 2013). In the context of novel environments for skill learning, such as laparoscopic surgery (FLS Trainer System Accessories, 2010), visuomotor learning requires perceptual learning (Kamat et al., 2022) engaging multiple brain regions (Dosenbach et al., 2007). This includes the dorsal attention network for visuospatial awareness and the salience network to focus attention on pertinent stimuli, such as visuomotor task-errors. Consequently, appropriate brain targets for tDCS necessitates consideration of relevant nodes within brain networks (Bressler and Menon, 2010) that are directly linked to the performance and learning of the specific visuomotor skill. In this study, we targeted two relevant brain nodes with tDCS, based on our published model (Walia et al., 2022a), to facilitate error-based learning that constitutes a fundamental mechanism in skill acquisition, encompassing error detection, correction, and subsequent performance adjustments (Seidler et al., 2013). Our application of tDCS in this study was guided by insights gained from analyzing error-related brain states by utilizing a combination of electroencephalography (EEG) and functional near-infrared spectroscopy (fNIRS) during laparoscopic suturing with intracorporeal knot tying task (Walia et al., 2022b). Fundamentals of Laparoscopic Surgery (FLS) suturing with intracorporeal knot tying is a complex motor task requiring high precision hand-eye coordination, depth perception from the 2D view (Dilley et al., 2020), and tool control for optimal performance (Hannah et al., 2022). Top panel of Figure 1 shows the six microstate prototypes discerned from the amalgamated EEG data encompassing both experts and novices performing the FLS complex task, elucidating 77.14% of the overall variance (Walia et al., 2022a). Figures 1A–E show color coded transition probabilities between microstate (MS) classes at the group level with Figure 1A showing that during the 10-s at the start of the FLS task in novices, Figure 1B show that during the 10-s at the start of the FLS task in experts, Figure 1C showing that during the 10-s in the error epoch in novices, and Figure 1D showing that during the 10-s in the error epoch in experts. Here, transition probabilities between MS classes during 10-s in the error epoch showed typical patterns when compared to 10-s at the start of the FLS task. Notably, differences in task-error perception and attention redirection for corrective actions were posited to vary between experts and novices (Walia et al., 2022a) where transition to MS 3 was highlighted in experts (Figure 1D) when compared to novices (Figure 1C) during the 10-s in the error epoch. Here, MS 3 exhibited topographical similarity to Bréchet et al. (2019) microstate D, with primary activity sources located bilaterally in the inferior frontal gyrus (IFG), dorsal anterior cingulate cortex (dACC), and the superior parietal lobule (SPL)/intraparietal sulcus (IPS). In addition, transition to MS 1 was highlighted in both experts (Figure 1D) and novices (Figure 1C) during the 10 s in the error epoch where MS 1 bears the closest topographical resemblance to Brechet and colleagues' microstate C (Bréchet et al., 2019), with sources situated bilaterally in the lateral portion of the parietal cortex, encompassing both the supramarginal gyrus (SMG) and angular gyrus (AG). Therefore, we proposed a model for task-error perception and attention redirection for corrective actions (Walia et al., 2022a) that posited attention driven by unexpected stimuli (errors), subserved by the engagement of the SMG and AG from ventral attention stream in both experts and novices – see Figure 1E. Then, in the experts, an adaptive control of goal-directed attention within the frontoparietal network system was posited, shown in Figure 1E, subserved by the activation of SPL and IPS due to the involvement of dorsal attention systems pertinent to the reorientation of visuospatial attention. Since the frontoparietal network, believed to support adaptive control, exhibited a 2.3-fold overrepresentation in the cerebellum compared to the cerebral cortex (Marek et al., 2018); therefore, cerebellar node was selected as one of the brain nodes for tDCS to facilitate error-based learning. Then, the other brain node for tDCS was selected as left ventrolateral prefrontal cortex (PFC) to facilitate subjective task-error awareness (Wessel, 2012) by modulating the left lateralized brain network for visuospatial attention (De Schotten et al., 2011; Vossel et al., 2014). Our model for task-error perception and attention redirection for corrective actions (Walia et al., 2022a) was supported recently by Choo et al. (2023) who identified contributions from the left lateral prefrontal, occipital, sensorimotor, and parietal channels for post-error adjustments. Specifically, the left lateral prefrontal channels played a predominant role in post-error adjustments, manifesting from around 900 ms into extended post-error periods (Choo et al., 2023). Consequently, modulating task-error perception and attention redirection for corrective actions using tDCS, assessed through portable brain imaging methods such as fNIRS (Gao et al., 2021a) and EEG (Ciechanski et al., 2019), and correlating these changes with tDCS modulated behavioral effects can offer mechanistic insights. In our multi-modal fNIRS-EEG approach to assess tDCS effects on the brain (Guhathakurta and Dutta, 2016), regularized temporally embedded canonical correlation analysis developed in our prior work (Walia et al., 2022a) was applied in the current study to find EEG band power changes that corresponded with the oxyhemoglobin (HbO) concentration changes from fNIRS data.

## 2 Materials and methods

### 2.1 Subjects and tasks

Sixteen healthy novices (age: 22–28 years) from medical/premedical student backgrounds, were recruited for the study (see Table 1 for details) that was approved by the Institutional Review Board of the University at Buffalo, USA. Four subjects withdrew from the full study (see Table 1) so twelve participants provided written informed consent to perform all the three sessions over2 months with a minimum 1 week gap between sessions. All subjects were instructed verbally with a standard set of instructions on completing the “FLS suturing with intracorporeal knot tying” task to the best of their ability. In this study, we only included novice subjects since upper limits in performance in expert surgeons may hinder additional enhancement through tDCS (Furuya et al., 2014).

**Table 1:** Subject demographics (four subjects in the grayed rows withdrew from the study).

**Participant**	**Age**	**Gender**	**Dominant hand**	**FLS experience**	**Progress**
P01	22	Male	Right	No	Completed
P02	26	Female	Right	No	Completed
P03	26	Female	Right	No	Completed
P04	28	Male	Right	No	Withdrew
P05	23	Female	Right	No	Completed
P06	23	Female	Right	No	Completed
P07	25	Female	Right	No	Withdrew
P08	24	Female	Left	No	Completed
P09	25	Female	Right	No	Completed
P10	25	Male	Right	No	Completed
P11	25	Female	Right	No	Withdrew
P12	24	Female	Right	No	Completed
P13	28	Female	Right	No	Completed
P14	23	Female	Right	No	Completed
P15	24	Female	Right	No	Withdrew
P16	25	Female	Right	No	Completed

**Figure 2:**
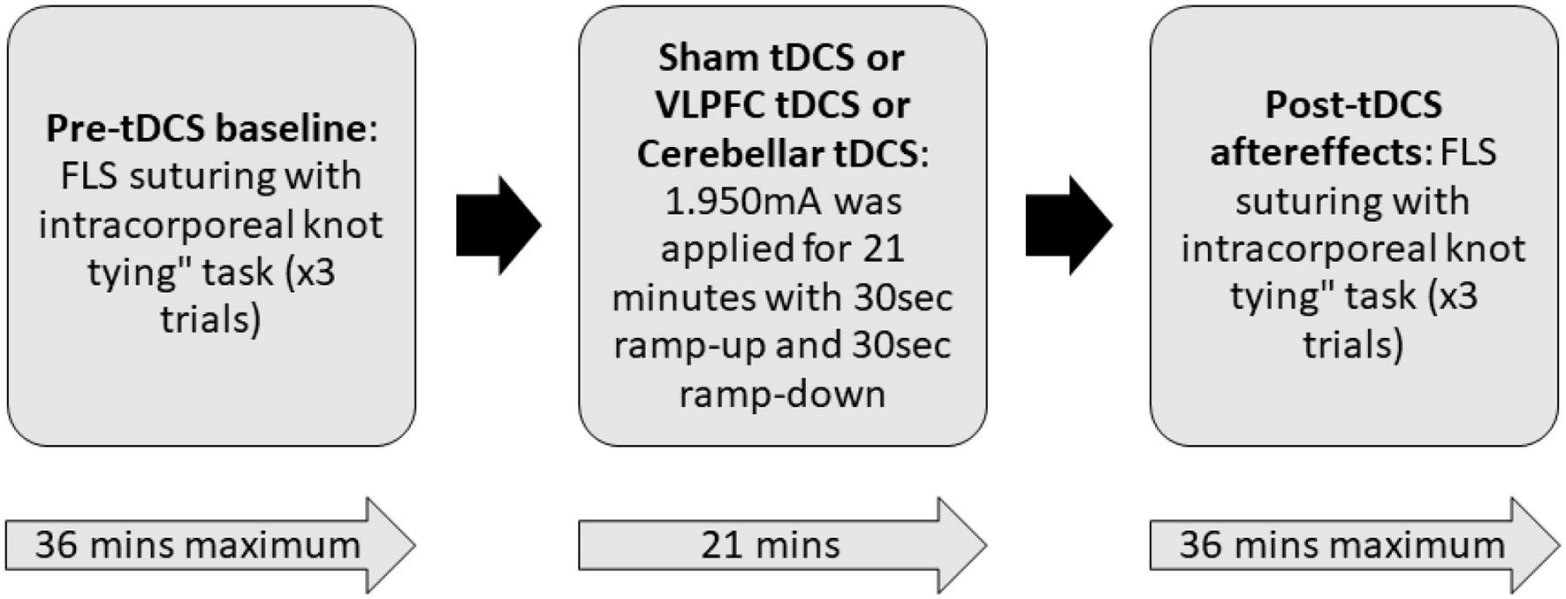
Block diagram of the experimental procedure. During the pre-tDCS and post-tDCS periods, each participant performed three repetitions of the FLS suturing with intracorporeal knot-tying task (maximum 10 min given) preceded by 2 min of rest period. During tDCS period, 1.950mA was applied for 21 min with 30 s ramp-up and 30 s ramp-down. In a randomized repeated measure design, subjects were allocated to three different sessions in random order that differed by the location of verum stimulation (left ventrolateral prefrontal cortex or right cerebellum) or sham stimulation. For sham tDCS, the current was ramped up in 30 s and then ramped down in 30 s to provide a sensation for the blinding effects.

Each participant completed three sessions that consisted of performing the intracorporeal suturing with knot tying task of the FLS curriculum in a standard FLS physical trainer box. Each session was divided into three parts: pre-tDCS baseline, tDCS application, and post-tDCS aftereffects, as shown in Figure 2. During the pre-tDCS and post-tDCS periods, each participant performed three repetitions of the FLS suturing with intracorporeal knot-tying task, i.e., a total of six repetitions per session. For the task, participants were provided with two laparoscopic needle drivers, one suturing scissors, and a needle with a suture of 15 cm in length. A Penrose drain with marked targets was placed on the Velcro strip inside the FLS Trainer Box. The task involves inserting the suture through two marks in a Penrose drain and then tying a double-throw knot followed by two single-throw knots using two needle graspers operated by both hands. The task starts when the subject picks up the suture and the needle driver on the “start” command (recorded along with the brain-behavior data) and ends when the subject cuts both ends of the suture, where the task completion is limited to 10 min (600 s). The task was repeated three times along with 2 min of the rest period, and the “start”'and the “stop” triggers for the task were registered with the data acquisition software. The experimenter labeled errors using the FLS box camera view of the error events at the “needle drop” and “incorrect needle insertion.”

### 2.2 Synchronized multimodal portable brain imaging

**Figure 3:**
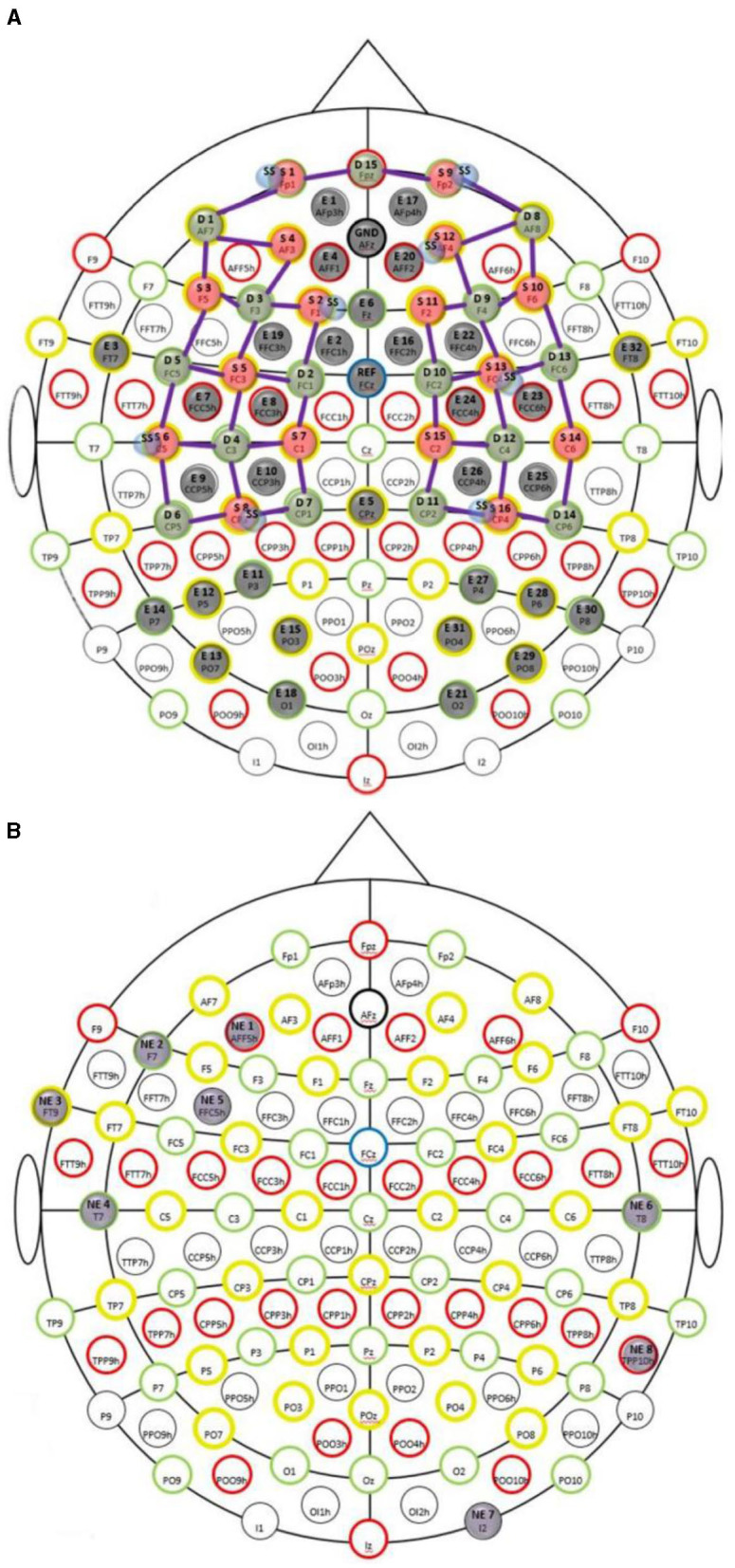
**(A)** Multi-modal brain imaging montage with fNIRS sources shown with red disks, fNIRS detectors shown with green disks, and the EEG electrodes shows with gray disks. **(B)** The eight Starstim 8 (Neuroelectrics, USA) electrodes are shown with gray disks where the labeled disks, NE1, NE5, and NE3, are anodes and NE2 is the cathode for VLPFC tDCS while NE8 is the anode and NE7 is the cathode for the CER tDCS.

A custom montage made of EEG electrodes and fNIRS optodes was used to record synchronized multimodal brain activation signals based on our prior works (Walia et al., 2022a). The 32-channel EEG signal was recorded using a wireless LiveAmp system (Brain Vision, United States). The EEG recordings were obtained at 500 Hz using active gel electrodes. Thirty-two channels of fNIRS signals and eight short-sequence channels were recorded at a 5 Hz sampling rate with NIRSPORT2 (NIRx, USA). A 1 Hz hardware trigger signal was implemented for fNIRS-EEG synchronization, aligning the multimodal data. The optical probes and EEG electrodes were mounted according to the standard 10–5 mount (see Figure 3A), and the automated anatomical labeling (AAL) (Rolls et al., 2020, p. 3) of the fNIRS probe (Aasted et al., 2015) is shown in Supplementary Table S1. Figure 3A shows dark green circles as EEG electrodes where ground and the reference are at AFz and FCz positions respectively. Then, the short separation detectors are attached with the sources which can be seen with small blue circles. The fNIRS source-detector channels are shown with the purple connecting lines. The probes were carefully placed on the subject's head to avoid hair interference and to avoid affecting the subject's mobility during the mobile brain behavior study. Supplementary Table S1 lists the labels for the cortical regions of interest based on the automatic anatomical labeling atlas (Rolls et al., 2020, p. 3) and the coordinate space of the Montreal Neurological Institute (Aasted et al., 2015). Figure 3B shows the stimulation electrodes placed over the left VLPFC (PFC tDCS) and right cerebellum (CER tDCS) for tDCS using Starstim 8 (Neuroelectrics, USA). The eight Starstim NG Pistim electrodes are shown with gray disks in Figure 3B where NE1, NE5, and NE3, are anode injecting 0.65 mA current each and NE2 is the cathode (return electrode for 1.950 mA) for VLPFC tDCS while NE8 is the anode injecting 1.950mA and NE7 is the cathode (return electrode for 1.950mA) for the CER tDCS. These electrode montages were selected from our prior works (Rezaee et al., 2021; Walia et al., 2021) that targeted left VLPFC (inferior frontal gyrus) or right cerebellum (Crus cerebri), demonstrated in Supplementary Figure S1, found from computational electric field modeling using default 6th gen MNI-152 head (https://nist.mni.mcgill.ca/mni-icbm152-non-linear-6th-generation-symmetric-average-brain-stereotaxic-registration-model/) (Huang et al., 2018). For neuromodulation, a total direct current of 1.950mA was applied for 21 min with 30 s ramp-up and 30 s ramp-down. Sessions differed by the location of verum stimulation (left VLPFC or right cerebellum) or sham tDCS, and the assignment order was randomized across participants. During Sham tDCS (SHM tDCS), the current was ramped up in 30 s and then ramped down in 30 s to provide the sensation for blinding effects.

### 2.3 fNIRS and EEG preprocessing

The simultaneously recorded EEG and fNIRS signals were pre-processed and analyzed offline. EEG signals were preprocessed using the open source EEGlab toolbox (https://sccn.ucsd.edu/eeglab/index.php). Specifically, the data was bandpass filtered (‘pop_eegfiltnew(EEG, 'locutoff',1,'hicutoff',40)') between 1 Hz and 40 Hz. Then, the clean_rawdata function (‘pop_clean_rawdata (EEG, 'FlatlineCriterion',5,'ChannelCriterion', 0.8, 'LineNoiseCriterion', 4,'Highpass','off', 'BurstCriterion', 20,'WindowCriterion','off', 'BurstRejection','off', 'Distance', 'Euclidian')') was used to clean the data and reject bad channels. The bad channels were interpolated using spherical splines (Perrin et al., 1989) followed by re-referencing to the global average (‘pop_reref(EEG, [],'interpchan',[])'). The clean_rawdata function also performed artifact subspace reconstruction (ASR). ASR is an automated method based on a user-specified parameter that can effectively remove transient EEG artifacts (Chang et al., 2020). We used a lower 'ChannelCriterion' parameter value of 0.8 while using other default parameters including 'BurstCriterion' parameter value of 20 where the optimal value is between 20 and 30 to balance between removing non-brain signals and retaining brain activities (Chang et al., 2020). Also, the Picard algorithm (Frank et al., 2022) for Independent Component Analysis (ICA) was applied in the EEGlab (‘pop_runica(ALLEEG, 'icatype','picard','concatcond','on','options',{'pca',-1})') to separate brain and non-brain related activities where the EEG in the same tDCS session was assumed by the clustering functions to have the same ICA component weights. Here, the “ Multiple Artifact Rejection Algorithm” (MARA) plug-in (Winkler et al., 2014) in the EEGlab with default settings was used to flag and remove artifactual EEG components.

Laplacian spatial filter (Kayser and Tenke, 2015) was applied in Matlab (https://psychophysiology.cpmc.columbia.edu/software/csdtoolbox/) to the preprocessed EEG data to reduce volume conduction from the subcortical sources while keeping the cortical sources that are postulated to better correspond with the hemodynamic response measured with fNIRS. The fNIRS data were processed using the standard open-source HOMER3 package (https://github.com/BUNPC/Homer3). The fNIRS preprocessing pipeline consisted of the following (Walia et al., 2022a): the intensity was converted to optical density, and then motion artifacts were detected and filtered with the help of the Savitzky-Golay filtering method (Jahani et al., 2018) with default parameters in HOMER3. The optical density was bandpass filtered in the neurovascular coupling band, 0.01Hz−0.1 Hz, and then converted to chromophore (HbO) concentration with a unit partial pathlength factor.

### 2.4 fNIRS-EEG joint analysis

In our prior work (Walia et al., 2022a), the correspondence between the changes in the fNIRS HbO changes and the EEG band power (1HZ−40Hz) was found based on the General Linear Model (GLM) and regularized canonical correlation analysis with temporal embedding in HOMER3 (von Lühmann et al., 2020). The evoked hemodynamic signal is typically reconstructed with a weighted set of temporal basis functions in HOMER3 (von Lühmann et al., 2020); however, in the current study, we reconstructed the fNIRS intensity measurements with an additional weighted set of multichannel EEG bandpower (1Hz−40Hz) signals to estimate neurovascular coupling effects (Walia et al., 2022a). Here, the design matrix consisted of all the regressors for GLM, namely, a consecutive sequence of Gaussian functions paramsBasis: (0.5 0.5) for the first 10 s of the task-onset hemodynamic response function, neurovascular coupling regressors from the regularized Canonical Correlation Analysis with Temporal Embedding (tCCA) of the EEG bandpower (1Hz−40Hz), nuisance regressors from the tCCA of the short separation (SS) fNIRS channels, and a 3rd order polynomial to model drift, that was solved with an ordinary least squares approach (Ye et al., 2009) for each regressor's contribution based on their coefficients (von Lühmann et al., 2020). Thus, the SS fNIRS signals provided the nuisance regressors for the systemic artifact (von Lühmann et al., 2020) while the EEG bandpower (‘bandpower' in Matlab) signals provided the neurovascular coupling regressors in the design matrix for GLM (‘hmrDeconvHRF_DriftSSnvc' solved the GLM matrix equation). Here, the HOMER3 function, “rtcca,” uses multimodal data to extract the nuisance and the neurovascular coupling regressors for the GLM-based noise reduction using tCCA regularized with shrinkage of covariance matrices (von Lühmann et al., 2020). Supplementary Figure S2 present the selection of parameters for the “rtcca” function (param.tau: temporal embedding parameter (lag in samples), param.NumOfEmb: number of temporally embedded copies, param.ct: correlation threshold) to find the neurovascular coupling regressors. Also, Supplementary Figure S2 shows an illustrative example of the neurovascular coupling regressors in the frequency domain where the top five regressors computed from the EEG bandpower (1Hz−40Hz) signals were used for solving the GLM matrix equation for the long separation (LS) fNIRS signals.

### 2.5 Statistical analysis of the EEG scalp topography response

Clustering of the Independent Component (ICs) was performed for the 0 s−20 s epoch at the start of the FLS task based on the power spectral parameters and the EEG scalp maps using affinity clustering and its default parameters in EEGlab (Bigdely-Shamlo et al., 2013). The event-related spectral perturbation (ERSP) was investigated using the time/frequency analysis in the EEGlab to best characterize the phase-incoherent FLS task event-related brain dynamics (Makeig, 1993). Here, the ERSP measured average dynamic changes in amplitude of the 1 – 40 Hz EEG frequency spectrum as a function of time relative to the start of FLS task event. Based on ERSP, an appropriate time window was selected to compare pre-tDCS (PRE) baseline scalp topography with the pos-tDCS (POS) changes using permutation statistics with FDR correction for multiple comparisons in the EEGlab.

### 2.6 Second level statistical analysis of the hemodynamic response after first level GLM

Each subject performed the FLS task before (PRE) and after (POST) tDCS conditions (CER, PFC, Sham), so, POST-PRE t-contrasts of the oxyhemoglobin (HbO) hemodynamic response from 0 s to 20 s at the start of the FLS task were analyzed using a mixed effects model (Santosa et al., 2018), “beta ~ −1 + cond + (1|subject)” where ‘cond' are the tDCS conditions. Here, the model has a term for each tDCS condition (CER, PFC, Sham) and the demographics variable “subject” is denoted as a random effect. The significance level was set at *q* < 0.05 for the Benjamini–Hochberg FDR-corrected *p*-values (termed *q*-values).

### 2.7 Statistical analysis of the behavioral response

The behavioral response to tDCS was measured based on POST-PRE change in the performance measures from the FLS curriculum for “FLS suturing with intracorporeal knot tying” task (Ritter and Scott, 2007). The post-tDCS change in the performance measures, namely, task time, deviation, incision gap, knot security, accuracy error, and the FLS score, from pre-tDCS baseline values was first tested for normality – see Supplementary Figure S3 using Kolmogorov-Smirnov and Shapiro-Wilk tests in IBM^®^ SPSS^®^ software platform. The normality tests rejected the null hypothesis at the default 5% significance level for most of the POST-PRE changes in the FLS performance measures – see Supplementary Figure S3. So, non-parametric Wilcoxon Rank sum test or Mann–Whitney *U*-test was applied to test the null hypothesis that POST-PRE changes in the FLS score for CER tDCS and Sham tDCS, PFC tDCS and Sham tDCS are samples from a continuous distributions with equal medians. We also computed *Post-hoc* power (Yuan and Maxwell, 2005) using G^*^Power software that refers to the retrospective power of an observed effect of verum tDCS vs. sham tDCS, determined by the sample size and parameter estimates derived from our experimental dataset.

## 3 Results

**Figure 4:**
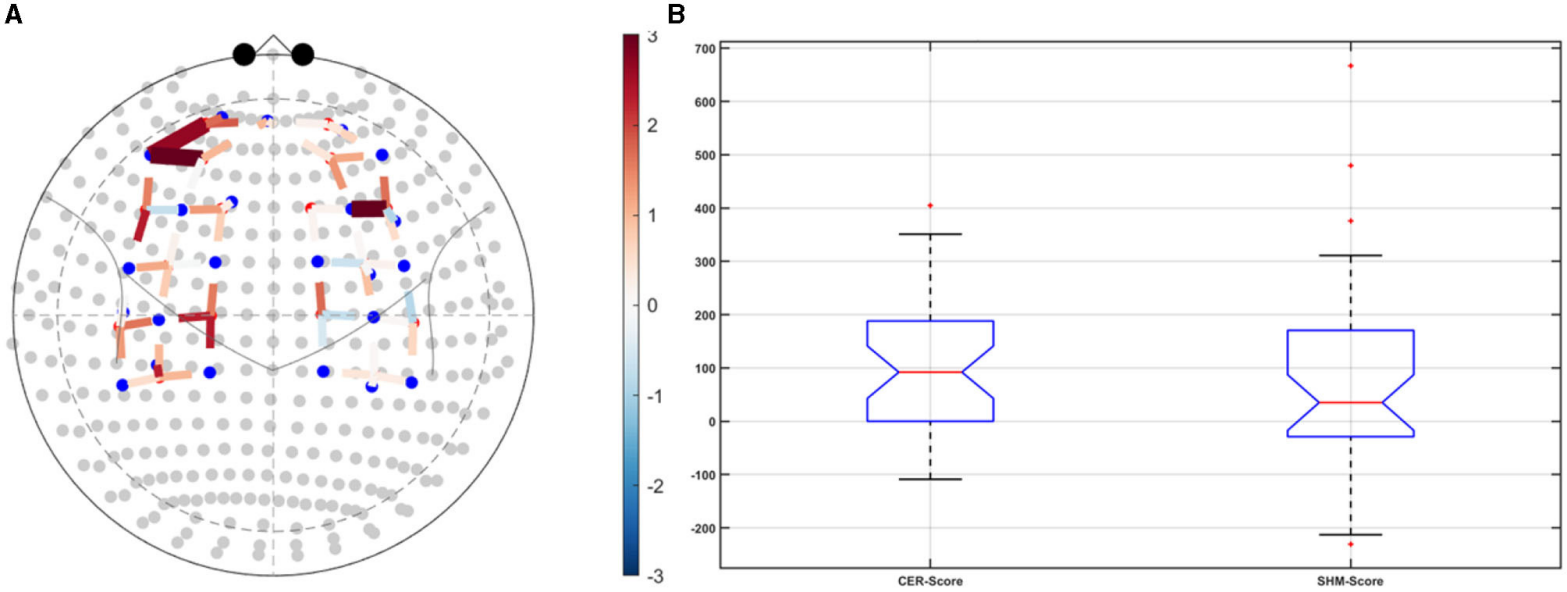
Right cerebellar (CER) transcranial direct current stimulation (tDCS) compared to Sham (SHM) tDCS. **(A)** t-statistics of the cerebellar tDCS modulation of the HbO hemodynamic response with the highest (*q* < 0.05) at the fNIRS channel (source 4 and detector 1) primarily overlying left middle frontal gyrus (AAL: 'frontal_mid_l') and fNIRS channel (source 10 and detector 9) primarily overlying right inferior frontal gyrus, triangular part (AAL: 'frontal_inf_tri_r'). **(B)** Post-tDCS (POST) – pre-tDCS (PRE) change in the FLS score for CER tDCS showed a significant (*p* < 0.05) difference from that for SHM tDCS.

**Figure 5:**
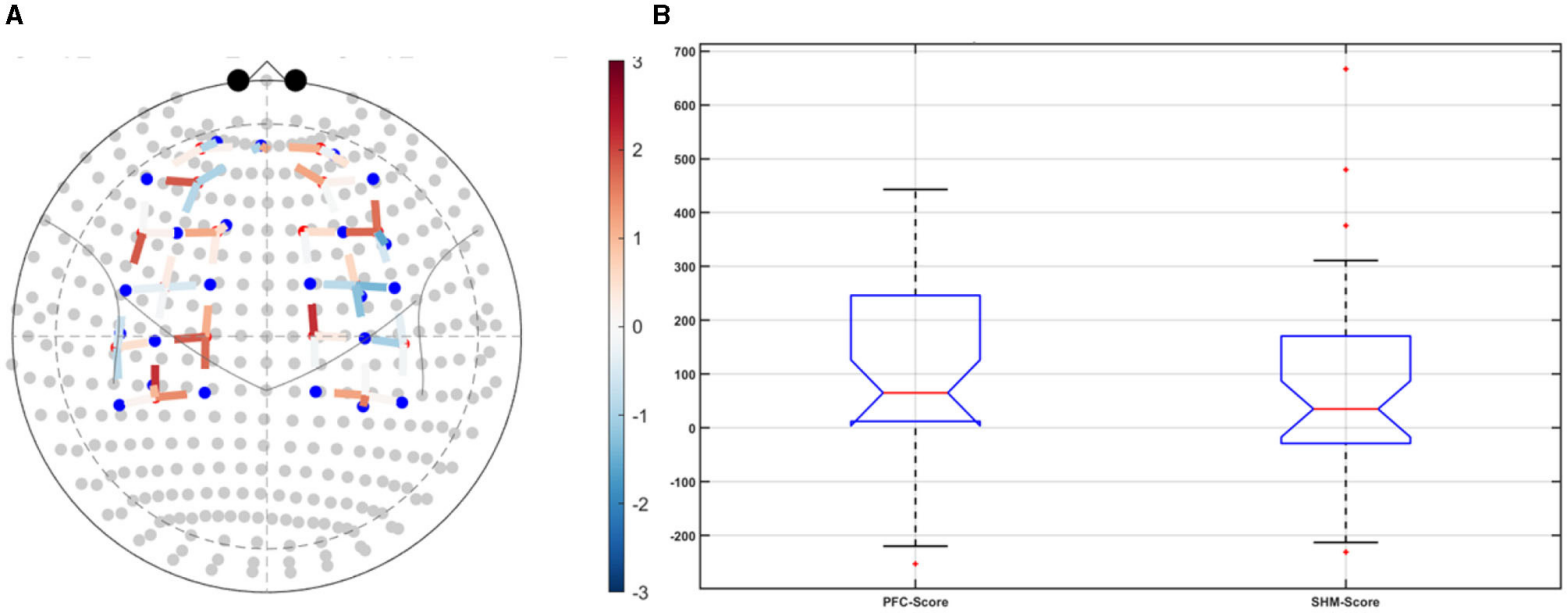
Left ventrolateral prefrontal Cerebellar (PFC) transcranial direct current stimulation (tDCS) compared to Sham (SHM) tDCS. **(A)** t-statistics of the PFC tDCS modulation of the HbO hemodynamic response. **(B)** Post-tDCS (POST) – pre-tDCS (PRE) change in the FLS score for PFC tDCS did not show a significant difference from that for SHM tDCS.

Post-tDCS change in the FLS performance measures, namely, task time, deviation, incision gap, knot security, accuracy error, from pre-tDCS baseline values are provided in the Supplementary Figure S4 while Supplementary Figure S5 presents the corresponding descriptive statistics table. A significant (*q* < 0.05) change in the oxyhemoglobin (HbO) hemodynamic response during the FLS task was found for change post-tDCS (POST) from pre-tDCS (PRE) for CER tDCS when compared to SHM tDCS – see Figure 4A. Then, CER tDCS effect on the change (POST-PRE) in the FLS score for CER tDCS when compared to SHM tDCS was significant (*p* < 0.05) – see Figure 4B. Figure 4A shows that the CER tDCS modulation of the HbO hemodynamic response was the highest (*q* < 0.05) at the fNIRS channel (source 4 and detector 1) primarily overlying left middle frontal gyrus (AAL: 'frontal_mid_l') and fNIRS channel (source 10 and detector 9) primarily overlying right inferior frontal gyrus, triangular part (AAL: 'frontal_inf_tri_r') based on the sensitivity (weights) of the fNIRS probe (Aasted et al., 2015) as listed in Table 2 (also see Supplementary Table S1). In contrast, the HbO hemodynamic response and the FLS behavioral effects (FLS score) of the PFC tDCS were not significant, as shown in Figure 5. Table 3 presents the descriptive statistics of post-tDCS change in the FLS score from pre-tDCS baseline values (ScoreDiff) for various interventions namely CER tDCS, PFC tDCS, SHM tDCS. *Post hoc* power analysis for normal distribution of FLS score (ScoreDiff for CER tDCS and PFC tDCS – see normality test results in Supplementary Figure S3) provided a *post-hoc* power of 0.05 for CER tDCS vs. PFC tDCS intervention.

**Table 2:** Sensitivity profile (weights) of the fNIRS montage (source – detector pairs) based on automated anatomical labeling (AAL) with the source-detector pairs with the highest (*q* < 0.05) cerebellar tDCS modulation of the HbO hemodynamic response in BOLD.

**Source**	**Detector**	**Sensitivity weight**	**AAL region**
3	1	0.002639855	‘frontal_inf_oper_l'
3	3	0.003595979	‘frontal_inf_oper_l'
3	5	0.993017537	‘frontal_inf_oper_l'
5	5	0.00074663	‘frontal_inf_oper_l'
1	1	0.013948443	‘frontal_inf_tri_l'
3	1	0.694668298	‘frontal_inf_tri_l'
3	3	0.041134755	‘frontal_inf_tri_l'
3	5	0.185502252	‘frontal_inf_tri_l'
**4**	**1**	**0.062855542**	**‘frontal_inf_tri_l'**
4	3	0.00189071	‘frontal_inf_tri_l'
9	8	0.009315436	‘frontal_inf_tri_r'
9	20	0.000189792	‘frontal_inf_tri_r'
10	8	0.585089378	‘frontal_inf_tri_r'
**10**	**9**	**0.052457539**	**‘frontal_inf_tri_r'**
10	13	0.011573739	‘frontal_inf_tri_r'
10	21	0.30435135	‘frontal_inf_tri_r'
12	8	0.033856984	‘frontal_inf_tri_r'
12	9	0.003165782	‘frontal_inf_tri_r'
1	1	0.526534988	‘frontal_mid_l'
1	15	0.005048587	‘frontal_mid_l'
1	16	0.087406083	‘frontal_mid_l'
2	2	7.73E-06	‘frontal_mid_l'
2	3	0.002154303	‘frontal_mid_l'
2	17	0.000235586	‘frontal_mid_l'
3	1	0.009652047	‘frontal_mid_l'
3	3	0.023100115	‘frontal_mid_l'
3	5	0.000903585	‘frontal_mid_l'
**4**	**1**	**0.332887408**	**‘frontal_mid_l'**
4	3	0.009660562	‘frontal_mid_l'
4	15	0.001713729	‘frontal_mid_l'
5	2	2.90E-05	‘frontal_mid_l'
5	3	0.000583378	‘frontal_mid_l'
5	5	8.29E-05	‘frontal_mid_l'
9	8	0.480119355	‘frontal_mid_r'
9	15	0.000591301	‘frontal_mid_r'
9	20	0.390412138	‘frontal_mid_r'
10	8	0.012227158	‘frontal_mid_r'
**10**	**9**	**0.002681805**	**‘frontal_mid_r'**
10	13	0.000102773	‘frontal_mid_r'
10	21	0.000919878	‘frontal_mid_r'
11	9	0.000311662	‘frontal_mid_r'
11	10	2.29E-05	‘frontal_mid_r'
12	8	0.091991418	‘frontal_mid_r'
12	9	0.01903687	‘frontal_mid_r'
12	15	0.000473541	‘frontal_mid_r'
13	9	0.000735614	‘frontal_mid_r'
13	10	2.29E-05	‘frontal_mid_r'
13	12	1.69E-05	‘frontal_mid_r'
13	13	0.000129123	‘frontal_mid_r'
13	22	0.000202524	‘frontal_mid_r'
14	12	2.14E-06	‘frontal_mid_r'
1	1	0.036332124	‘frontal_sup_l'
1	15	0.463426394	‘frontal_sup_l'
1	16	0.377885587	‘frontal_sup_l'
2	2	9.84E-06	‘frontal_sup_l'
2	3	0.000383442	‘frontal_sup_l'
2	17	0.000310116	‘frontal_sup_l'
**4**	**1**	**0.009695995**	**‘frontal_sup_l'**
4	3	0.000904605	‘frontal_sup_l'
4	15	0.111049264	‘frontal_sup_l'
5	2	2.63E-06	‘frontal_sup_l'
1	15	3.74E-06	‘frontal_sup_r'
9	8	0.001976993	‘frontal_sup_r'
9	15	0.708585009	‘frontal_sup_r'
9	20	0.046833513	‘frontal_sup_r'
11	9	4.05E-05	‘frontal_sup_r'
11	10	9.49E-06	‘frontal_sup_r'
12	8	0.001982347	‘frontal_sup_r'
12	9	0.000120626	‘frontal_sup_r'
12	15	0.240442543	‘frontal_sup_r'
13	10	5.28E-06	‘frontal_sup_r'
6	4	0.116188887	‘parietal_inf_l'
6	5	0.116037034	‘parietal_inf_l'
6	6	0.382524029	‘parietal_inf_l'
6	18	0.38525005	‘parietal_inf_l'
14	12	0.948731	‘parietal_inf_r'
14	14	0.051269	‘parietal_inf_r'
14	12	1	‘parietal_sup_r'
3	5	0.184021254	‘postcentral_l'
5	4	0.021004293	‘postcentral_l'
5	5	0.354191037	‘postcentral_l'
6	4	0.007334286	‘postcentral_l'
6	5	0.284621985	‘postcentral_l'
6	18	0.148827145	‘postcentral_l'
10	13	0.126808776	‘postcentral_r'
13	12	0.005053639	‘postcentral_r'
13	13	0.127123934	‘postcentral_r'
13	22	0.003028674	‘postcentral_r'
14	12	0.024951637	‘postcentral_r'
14	13	0.713033339	‘postcentral_r'
3	3	0.008656183	‘precentral_l'
3	5	0.86776482	‘precentral_l'
5	2	0.003898449	‘precentral_l'
5	3	0.013912468	‘precentral_l'
5	4	0.00081039	‘precentral_l'
5	5	0.10106652	‘precentral_l'
6	5	0.00389117	‘precentral_l'
**10**	**9**	**0.002589612**	**‘precentral_r'**
10	13	0.835729571	‘precentral_r'
10	21	0.050975773	‘precentral_r'
13	9	0.001950371	‘precentral_r'
13	12	0.002583249	‘precentral_r'
13	13	0.077745113	‘precentral_r'
13	22	0.021469151	‘precentral_r'
14	13	0.006957159	‘precentral_r'
6	4	0.002920897	‘supramarginal_l'
6	5	0.424325487	‘supramarginal_l'
6	6	0.041415336	‘supramarginal_l'
6	18	0.531338281	‘supramarginal_l'
14	12	0.078410233	‘supramarginal_r'
14	13	0.859373704	‘supramarginal_r'
14	14	0.062216063	‘supramarginal_r'

**Table 3:** Descriptive statistics of post-tDCS change in the FLS score from pre-tDCS baseline values (ScoreDiff) for various interventions namely CER tDCS, PFC tDCS, and SHM tDCS.

**ScoreDiff**	**CER**	**Mean**		**106.2222**	**21.20943**
		95% confidence interval for mean	Lower bound	63.1648	
			Upper bound	149.2797	
		5% trimmed mean		101.8272	
		Median		92.0000	
		Variance		16194.235	
		Std. deviation		127.25657	
		Minimum		−109.00	
		Maximum		405.00	
		Range		514.00	
		Interquartile range		206.00	
		Skewness		0.482	0.393
		Kurtosis		−0.401	0.768
	**PFC**	**Mean**		**108.61111**	**28.91614**
		95% confidence interval for mean	Upper bound	49.9082	
			Lower bound	167.3140	
		5% trimmed mean		109.3086	
		Median		65.0000	
		Variance		30101.159	
		Std. deviation		173.49686	
		Minimum		−253.00	
		Maximum		443.00	
		Range		696.00	
		Interquartile range		259.50	
		Skewness		0.192	0.393
		Kurtosis		−0.318	0.768
	**SHM**	**Mean**		**75.6667**	**30.38836**
		95% confidence interval for mean	Lower bound	13.9760	
			Upper bound	137.3583	
		5% trimmed mean		64.0247	
		Median		35.0000	
		Variance		33244.286	
		Std. deviation		182.33016	
		Minimum		−231.00	
		Maximum		667.00	
		Range		898.00	
		Interquartile Range		210.25	
		Skewness		1.227	0.393
		Kurtosis		2.355	0.768

**Figure 6:**
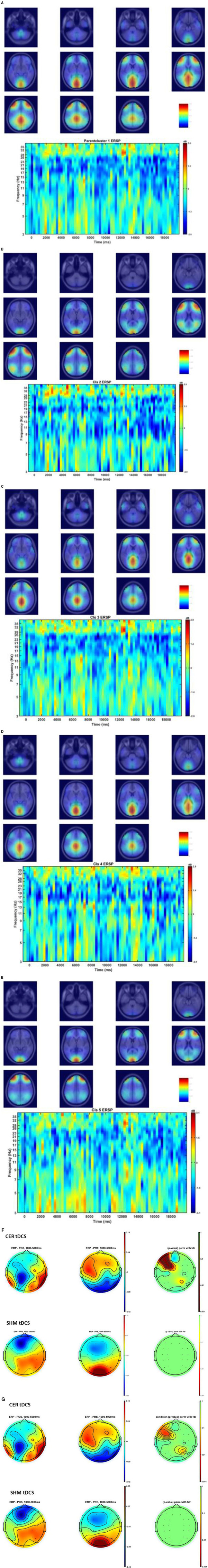
**(A**–**E)** Shows the Independent Components (IC) clusters for the CER tDCS session (left panel) and the cluster's averaged group dipoles density at the pre-tDCS (PRE) baseline for the FLS task epoch (right top panel). Also, shown is the cluster's Event-Related Spectral Dynamics (ERSP) at the baseline (PRE) for the FLS task epoch (right bottom panel). **(A)** parent IC cluster 1, **(B)** IC cluster 2, **(C)** IC cluster 3, **(D)** IC cluster 4, **(E)** IC cluster 5. **(F)** Averaged scalp topographies (1Hz to 40Hz) from 1 to 5 secs of the FLS task for the baseline (PRE) and post-stimulation (POS) for CER tDCS and SHM tDCS, and the associated FDR corrected *p*-value map to compare POS and PRE conditions (extreme right). **(G)** Averaged scalp topographies (1Hz to 8Hz) from 1 s to 5 s of the FLS task for the baseline (PRE) and post-stimulation (POS) for CER tDCS and SHM tDCS, and the associated FDR corrected *p*-value map to compare POS and PRE conditions (extreme right).

**Figure 7:**
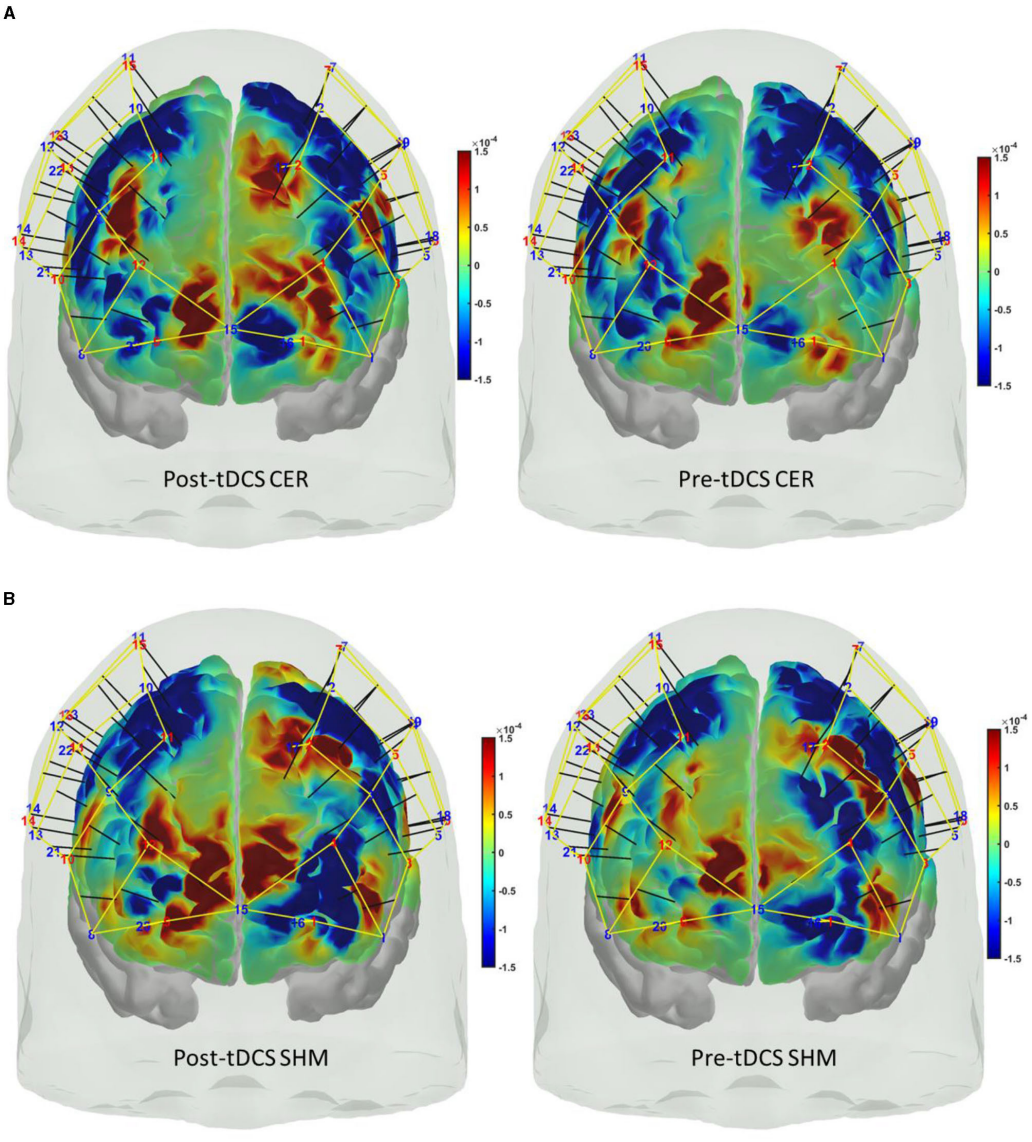
Cortical activation based on the oxyhemoglobin (HbO) hemodynamic response at the prefrontal areas at the start (5 s to 15 s) of the FLS task during post-tDCS (left panel) as well as pre-tDCS (right panel) baseline for **(A)** cerebellar (CER) tDCS, **(B)** sham (SHM) tDCS.

Figures 6A–E show the Independent Components (IC) clusters for the CER tDCS session that are averaged group dipoles with density at the baseline (PRE) for the FLS task epoch. ERSP at the baseline (PRE) for the FLS task epoch (right bottom panel) indicated activity starting after 1 s where the averaged 1Hz−40Hz scalp topography changes post-tDCS (POS) reached significance (*p* < 0.05, FDR corrected) within the 5 s, as shown in the Figure 6F. Figure 6G shows the averaged 1HZ−8Hz scalp topography changes post-tDCS (POS) from baseline that also reached significance (*p* < 0.05, FDR corrected) within the 5 s. Then, the hemodynamic response due to neurovascular coupling from 5 s to 15 s based on the cortical HbO activation (Aasted et al., 2015) at the start of the FLS task post-tDCS are shown in Figure 7 for CER and SHM tDCS. Supplementary Figure S6 shows the corresponding comparison between PFC tDCS (see Supplementary Figure S6a) and for SHM tDCS (see Supplementary Figure S6b).

## 4 Discussion

Our results showed a significant effect of cerebellar (CER) tDCS on POST-PRE HbO hemodynamic response during 0 s−20 s of the FLS suturing with intracorporeal knot tying task when compared to sham (SHM) tDCS. This significant response was primarily (highest optode sensitivity weight) at the left middle frontal gyrus and right inferior frontal gyrus (triangular part) which was expected due to CER tDCS targeting the posterior lobules [see Supplementary Figure S1, also shows the regions of the human cerebellum with color coding their functional connectivity to 7 major networks in the cerebrum (Buckner et al., 2011)] (Rezaee et al., 2021), in particular Crus I and II, that has projections to the prefrontal cortex (however, spared anatomical connections to motor cortex) (Buckner et al., 2011). Table 2 shows AAL regions, 'frontal_inf_tri_l,' 'frontal_inf_tri_r,' 'frontal_mid_l,' 'frontal_mid_r,' 'frontal_sup_l,' 'precentral_r,' that are all underlying the significant fNIRS optode pairs (source 4 and detector 1, source 10 and detector 9) with different sensitivity weights. CER tDCS facilitated FLS task related ERSP difference seems to be driven by the left prefrontal scalp topography – see the associated *p*-value map at the extreme right for CER tDCS (Figure 6F) when compared to SHM tDCS (Figure 6G). Here, right CER tDCS facilitated the left prefrontal activation ('frontal_inf_tri_l,' 'frontal_mid_l,' 'frontal_sup_l') at the start of the FLS task that was found underlying the fNIRS source 4 and detector 1, as evident in Figure 7A, when comparing post-tDCS cortical activation from pre-tDCS baseline.

CER tDCS modulation can be associated with the frontoparietal facilitation (Lückmann et al., 2014) of higher cognitive functions (du Boisgueheneuc et al., 2006) and proactive control (Hu et al., 2016) respectively – both relevant in error-based learning known to be associated with cerebellum. Here, frontal and parietal regions are attributed roles in attentional mechanisms, suggesting their involvement in allocating attentional resources to perceptual representations necessary at the beginning of the FLS task learning [perceptual learning (Kamat et al., 2022)]. We found that the CER tDCS effects on the behavior was significant (*p* < 0.05), as shown in the Figure 4B, with an improved POST-PRE change in the FLS score when compared to sham tDCS. Then, left ventrolateral prefrontal (PFC) tDCS modulation was postulated to facilitate task-error perception; however, that did not have a significant effect on the behavior when compared to sham tDCS, as shown in the Figure 5B. Also, left PFC tDCS effect on the HbO hemodynamic response was not significantly different from sham tDCS (see Figure 5A). Supplementary Figure S3 shows PFC post-tDCS effect at the left prefrontal areas ('frontal_mid_l,' 'frontal_sup_l') underlying fNIRS optode pair, source 4 and detector 15, when compared to pre-tDCS baseline. However, left PFC tDCS was postulated to facilitate the perception of the task-error but was not sufficient to facilitate brain activation at the start of the FLS task when compared to sham tDCS. Here, CER tDCS facilitation of the prediction model of the sensory outcome of action is postulated to be more important in skill-naïve novices involved in perceptual learning (Kamat et al., 2022) when compared to task-error perception with sensory error prediction model not available in skill-naïve (Tsay et al., 2022). Therefore, we postulate based on our study results that CER tDCS facilitation of sensory outcome prediction of action was helpful in novices who are FLS skill-naive (Crihfield et al., 2023) and are at the start of the skill learning (Mizuguchi et al., 2018). As the novices gain skill, the left PFC tDCS may facilitate task-error perception to modulate implicit motor recalibration driven together with sensory prediction errors (Tsay et al., 2022). Nevertheless, the effect size of CER tDCS vs. PFC tDCS was relatively small (*post-hoc* power of 0.05), and both verum tDCS interventions improved FLS score from SHM tDCS's median ScoreDiff of 35 to left PFC tDCS's median ScoreDiff of 65 and right CER tDCS's median ScoreDiff of 92 (see Table 3), i.e., 85% improvement for left PFC tDCS and 162% improvement for right CER tDCS from SHM tDCS's median ScoreDiff. Notably, bilateral prefrontal cortex (anode over F3, cathode over F4) tDCS (Ashcroft et al., 2020; Patel et al., 2021) resulted in a POST-PRE performance score difference in open knot-tying task of 17 for verum tDCS and −17.5 for sham tDCS.

In the current study, right CER tDCS and the related HbO activity at the left inferior and middle frontal gyrus (AAL: 'frontal_mid_l') during the start of the FLS task can be related to the encoding of sensory prediction errors (Schlerf et al., 2012; Ficco et al., 2021), that can be postulated to interact with the task-errors (Tsay et al., 2022) at later stages in skill learning for an improved FLS score. Then, the activation of the right inferior frontal gyrus, triangular part (AAL: 'frontal_inf_tri_r') can be related to predictive error related stopping specifically under situations with high uncertainty (Levy and Wagner, 2011); therefore, a possible coordination with the HbO activity at the left inferior and middle frontal gyrus for error-related perception action coupling can be postulated. Indeed, CER tDCS improved FLS accuracy (see descriptive statistics on ErrorDiff in Supplementary Figure S5) that trended toward improvement better than SHM and PFC tDCS but was not found to be statistically significant (*p* < 0.05). Here, future studies can investigate transcranial alternating current stimulation of the cortico-cerebello-thalamo-cortical loop that has been shown feasible in other studies (Singh et al., 2021; Walia et al., 2022c).

Our results on tDCS-modulated brain activation and behavioral effects aligned with numerous functional magnetic resonance imaging (fMRI) and fNIRS studies that have been published on skill learning (Roberts et al., 2006; Leff et al., 2007, 2008a,b; Wanzel et al., 2007; Ohuchida et al., 2009; Khoe et al., 2020; Gao et al., 2021a,b). Published fMRI studies have shown that a large-scale brain network encodes motor learning and transfer of learning from related past experiences (Heitger et al., 2012; Gerraty et al., 2014). The prefrontal cortex has been found to integrate the information necessary for action generation and perception (Raos and Savaki, 2017) relevant to error processing. Here, successful skill acquisition leads to an internal forward model (Wolpert et al., 1998) that can simulate the perceptual consequences of planned and executed motor commands. An intact action-perception coupling has been shown to depend on the integrity of the cerebellum (Christensen et al., 2014) that underpins the internal model (Ebner, 2013) and error-based learning (Popa and Ebner, 2019). Error-based sensorimotor learning also involves other areas of the brain, including the parietal cortex, striatum, and anterior cingulate cortex (Seidler et al., 2013). Furthermore, the cingulate and pre-supplementary motor areas are the generator sites of error-related negativity that are time-locked to an erroneous response (Seidler et al., 2013). Here, the role of bilateral inferior frontal junction (IFJ) activation and their coordination in task-error perception action coupling, i.e., an interaction between top-down behavioral goals and bottom-up capture of relevant stimulus, needs further investigation since IFJ responds generally to the onset of behaviorally relevant stimuli (Levy and Wagner, 2011), e.g., task-error in the current study. Indeed, early efferent error prediction can lead to preemptive adjustments, e.g., skilled typists execute errors with lighter keystrokes than novices. Then, fMRI studies can show additional activation of deeper brain structures (Roberts et al., 2006) that cannot be measured using fNIRS. Here, it is known from skill training with fMRI studies that the hierarchy of cognitive control shows a rostrocaudal axis in the frontal lobe, where a shift from posterior to anterior is postulated to mediate progressively abstract higher-order control. In the current study, CER tDCS effect on FLS task related cortical activation included region 6vr+ anterior to the map of premotor area 6 (Thomas Yeo et al., 2011), i.e., the microstructural border between the motor and the cognitive domain (Geyer, 2004; Amunts et al., 2010). The region 6vr+ has greatest connectivity between the extent (as percentage) of the cerebral surface area and the cerebellar gray matter volume (Buckner et al., 2011), which needs future investigation based on fNIRS directed functional connectivity [specifically, subserved by ventral superior longitudinal fascicle–SLF III (Kamat et al., 2022)] vis-à-vis error-related perception action coupling. Also, tDCS effects on the neurovascular unit (Arora and Dutta, 2023) needs investigation where a POS-PRE decrease in the left prefrontal scalp EEG activity (within 1Hz−40Hz and 1 s−5 s at the start of the FLS task) was found to be related to an increase in the left prefrontal HbO cortical activation (from 5 s to 15 s) – see Figures 6F, 7A. Notably, the left prefrontal scalp ERSP changes were partly driven by the EEG frequencies below 8Hz [e.g., theta activity as mechanism of cognitive control (Cavanagh and Frank, 2014)] as seen in the Figures 6E, G.

The limitations of the current study methods include small subject size and portable fNIRS with limited spatial and depth sensitivity (Strangman et al., 2013) that could provide a partial view of brain activation. High-density EEG can be provide deeper localization (Michel and Brunet, 2019) and can be combined with fNIRS for better depth sensitivity. Therefore, the limitations include a low-density fNIRS and EEG sensor montage that limited spatial resolution and depth sensitivity necessary to capture the complete hierarchy of the task related brain areas. Another limitation was unequal gender balance (two males, 10 females) in the small number of participants with only one female subject with left hand dominance; however, removal of those subjects did not affect the trend of the brain imaging and FLS performance measure outcomes to tDCS interventions. In conclusion, our current brain-behavior study provided mechanistic insights into the right cerebellar tDCS facilitated activation of the bilateral prefrontal areas at the start of the FLS suturing with intracorporeal knot-tying task that was related to a significantly improved FLS performance score when compared to sham tDCS. In contrast, left ventrolateral prefrontal cortex tDCS failed to improve FLS performance score significantly when compared to sham tDCS although there was a trend toward improvement. Importantly, in the current study we did not study tDCS effects longitudinally during skill training, in correspondence with the skill retention study by Gao and colleagues (Gao et al., 2021a), which may be helpful in delineating CER tDCS vs. PFC tDCS effects in the future.

## Data availability statement

The data that support the findings of this study are available on request from the corresponding author.

## Ethics statement

The studies involving humans were approved by the Institutional Review Board of the University at Buffalo, USA. The studies were conducted in accordance with the local legislation and institutional requirements. The participants provided their written informed consent to participate in this study.

## Author contributions

PW: data curation, investigation, experiments, and writing–draft preparation. YF: data curation, investigation, and experiments. JN, SS, and XI: supervision. SD and LC: supervision and writing—reviewing and editing. AD: study conceptualization, methodology, and writing—reviewing and editing. All authors contributed to the article and approved the submitted version.
