# Supplementary material for: Brain-behavior analysis of transcranial direct current stimulation effects on a complex surgical motor task
Source: Front Neuroergon. 2024 Jan 9;4:1135729. doi: 10.3389/fnrgo.2023.1135729 (PMC10790853; doi:10.3389/fnrgo.2023.1135729)
Supplement: Supplementary file 1 [file Data_Sheet_1.pdf]

Supplementary Tables

Table S1: Automated anatomical labeling (AAL) and Montreal Neurological Institute (MNI) coordinates of the cortical areas underlying the fNIRS channels (source # – detector # pair) when projected in the cortex in AtlasViewer using its default head model (Aasted et al., 2015).

Source #	Detector #	Channel Coordinates (MNI)	AAL label names	Regions
1	1	-30 56 -7	Frontal_Inf_Orb_L	Inferior frontal gyrus, orbital part Left hemisphere
1	15	-9 64 -8	Frontal_Sup_Orb_L	Superior frontal gyrus, orbital part Left hemisphere
1	16	-11 57 -5	Frontal_Sup_Orb_L	Superior frontal gyrus, orbital part Left hemisphere
2	2	-17 26 48	Frontal_Sup_L	Superior frontal gyrus, dorsolateral Left hemisphere
2	3	-21 34 28	Frontal_Mid_L	Middle frontal gyrus Left hemisphere
2	17	-13 45 38	Frontal_Sup_L	Superior frontal gyrus, dorsolateral Left hemisphere
3	1	-35 45 -2	Frontal_Inf_Orb_L	Inferior frontal gyrus, orbital part Left hemisphere
3	3	-30 33 20	Frontal_Mid_L	Middle frontal gyrus Left hemisphere
3	5	-36 8 13	Frontal_Inf_Oper_L	Inferior frontal gyrus, opercular part Left hemisphere
4	1	-38 55 -2	Frontal_Mid_Orb_L	Superior frontal gyrus, medial orbital Left hemisphere
4	3	-37 48 14	Frontal_Inf_Tri_L	Inferior frontal gyrus, triangular part Left hemisphere
4	15	-16 61 0	Frontal_Sup_Orb_L	Superior frontal gyrus, orbital part Left hemisphere
5	2	-47 12 53	Frontal_Mid_L	Middle frontal gyrus Left hemisphere
5	3	-47 24 31	Frontal_Mid_L	Middle frontal gyrus Left hemisphere
5	4	-47 -9 41	Postcentral_L	Postcentral gyrus Left hemisphere
5	5	-58 11 28	Precentral_L	Precental gyrus Left hemisphere
6	4	-46 -23 34	Postcentral_L	Postcentral gyrus Left hemisphere

6	5	-57 -11 15	Temporal_Sup_L	Superior temporal gyrus Left hemisphere
6	6	-37 -34 16	Rolandic_Oper_L	Rolandic operculum Left hemisphere
6	18	-63 -19 18	SupraMarginal_L	Supramarginal gyrus Left hemisphere
7	2	-27 -4 63	Frontal_Sup_L	Superior frontal gyrus, dorsolateral Left hemisphere
7	4	-50 -22 63	Postcentral_L	Postcentral gyrus Left hemisphere
7	7	-18 -34 57	Postcentral_L	Postcentral gyrus Left hemisphere
8	4	-46 -30 47	Postcentral_L	Postcentral gyrus Left hemisphere
8	6	-52 -49 34	SupraMarginal_L	Supramarginal gyrus Left hemisphere
8	7	-32 -47 54	Parietal_Inf_L	Inferior parietal, but supramarginal and angular gyri Left hemisphere
8	19	-29 -43 40	Parietal_Inf_L	Inferior parietal, but supramarginal and angular gyri Left hemisphere
9	8	41 57 -8	Frontal_Mid_Orb_R	Middle frontal gyrus, orbital part Right hemisphere
9	15	15 67 -11	Frontal_Sup_Orb_R	Superior frontal gyrus, orbital part Right hemisphere
9	20	21 54 -1	Frontal_Sup_R	Superior frontal gyrus, dorsolateral Right hemisphere
10	8	47 47 -2	Frontal_Inf_Orb_R	Inferior frontal gyrus, orbital part Right hemisphere
10	9	51 37 18	Frontal_Inf_Tri_R	Inferior frontal gyrus, triangular part Right hemisphere
10	13	55 14 10	Frontal_Inf_Oper_R	Inferior frontal gyrus, opercular part Right hemisphere
10	21	44 19 8	Frontal_Inf_Tri_R	Inferior frontal gyrus, triangular part Right hemisphere
11	9	23 36 32	Frontal_Sup_R	Superior frontal gyrus, dorsolateral Right hemisphere

11	10	33 34 56	Frontal_Sup_R	Superior frontal gyrus, dorsolateral Right hemisphere
12	8	42 53 -1	Frontal_Mid_Orb_R	Middle frontal gyrus, orbital part Right hemisphere
12	9	49 56 16	Frontal_Mid_R	Middle frontal gyrus Right hemisphere
12	15	18 62 -1	Frontal_Sup_Orb_R	Superior frontal gyrus, orbital part Right hemisphere
13	9	53 24 32	Frontal_Inf_Tri_R	Inferior frontal gyrus, triangular part Right hemisphere
13	10	45 9 47	Precentral_R	Precental gyrus Right hemisphere
13	12	57 -7 46	Precentral_R	Precental gyrus Right hemisphere
13	13	58 9 26	Precentral_R	Precental gyrus Right hemisphere
13	22	48 6 38	Precentral_R	Precental gyrus Right hemisphere
14	12	63 -20 36	SupraMarginal_R	Supramarginal gyrus Right hemisphere
14	13	43 -8 18	Insula_R	Insula Right hemisphere
14	14	46 -35 18	Temporal_Sup_R	Superior temporal gyrus Right hemisphere
15	10	36 -7 64	Frontal_Sup_R	Superior frontal gyrus, dorsolateral Right hemisphere
15	11	39 -38 76	Postcentral_R	Postcentral gyrus Right hemisphere
15	12	41 -22 52	Precentral_R	Precental gyrus Right hemisphere
16	11	42 -49 57	Parietal_Sup_R	Superior parietal gyrus Right hemisphere
16	12	51 -33 49	SupraMarginal_R	Supramarginal gyrus Right hemisphere
16	14	45 -46 34	Angular_R	Angular gyrus Right hemisphere
16	23	35 -46 42	Angular_R	Angular gyrus Right hemisphere

Supplementary Figures

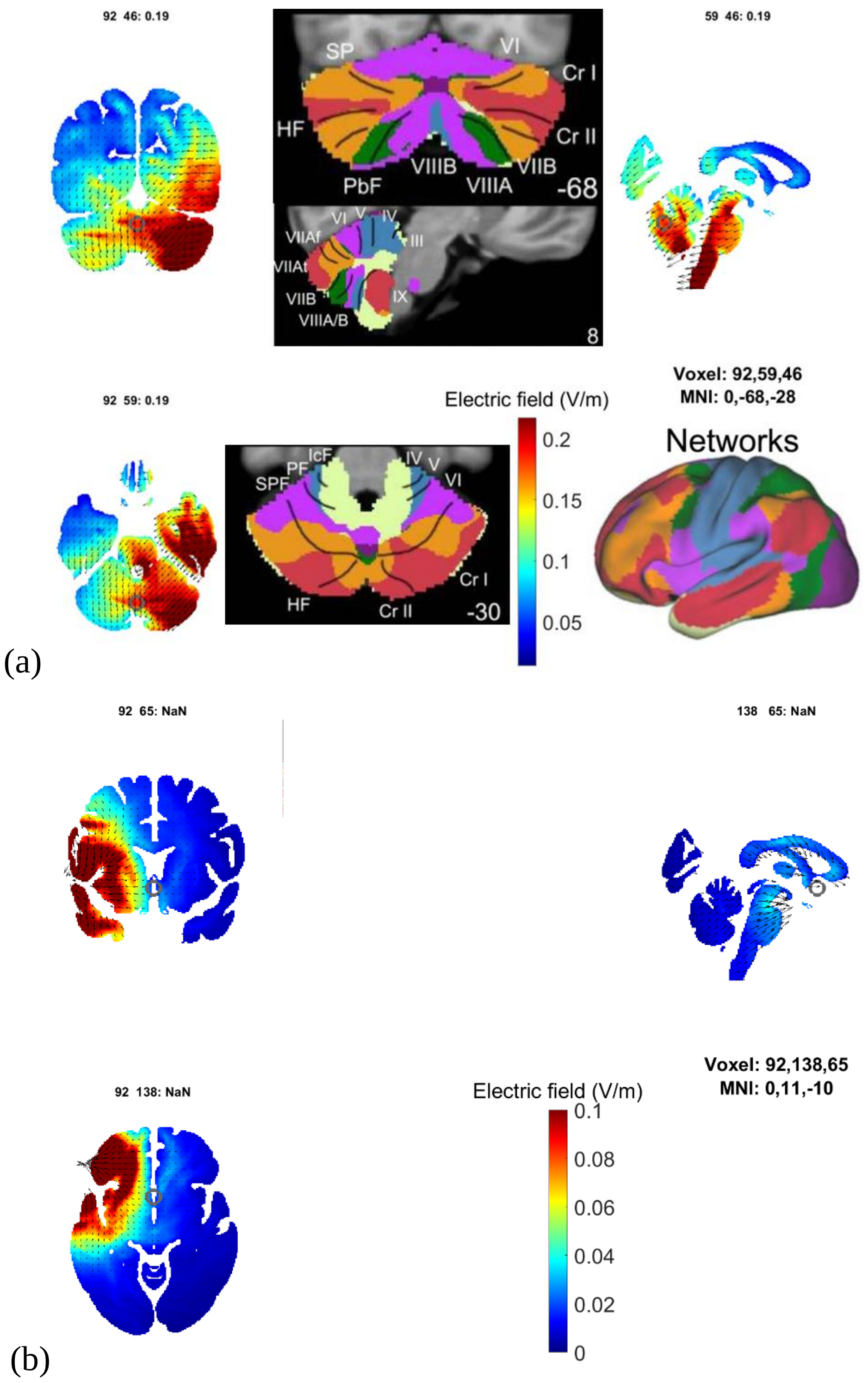

Figure S1: Computational modeling of the electric field distribution due to transcranial direct current stimulation (tDCS). (a) Right cerebellar tDCS along with the regions of the human cerebellum with the functional connectivity to 7 major networks in the cerebrum from Buckner et al. J Neurophysiol. 2011 Nov;106(5):2322-45. (b) Left ventrolateral prefrontal cortex tDCS.

(a)

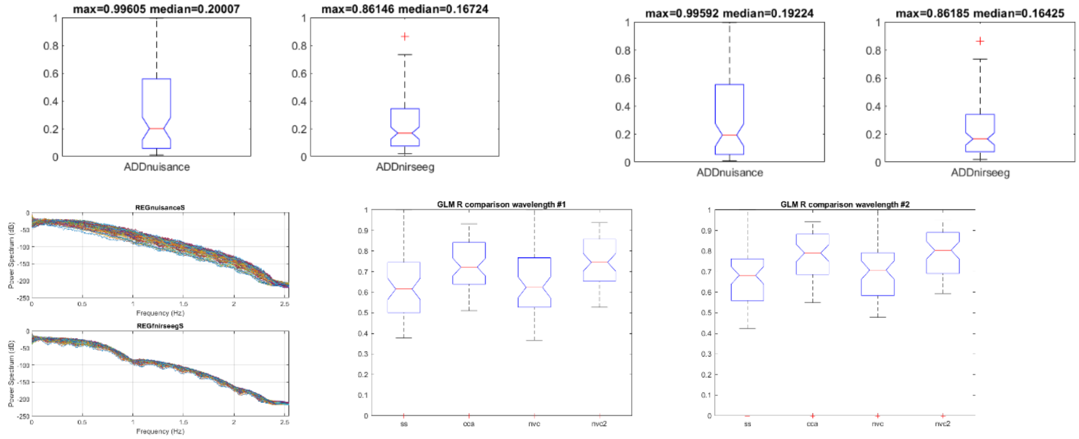

(b)

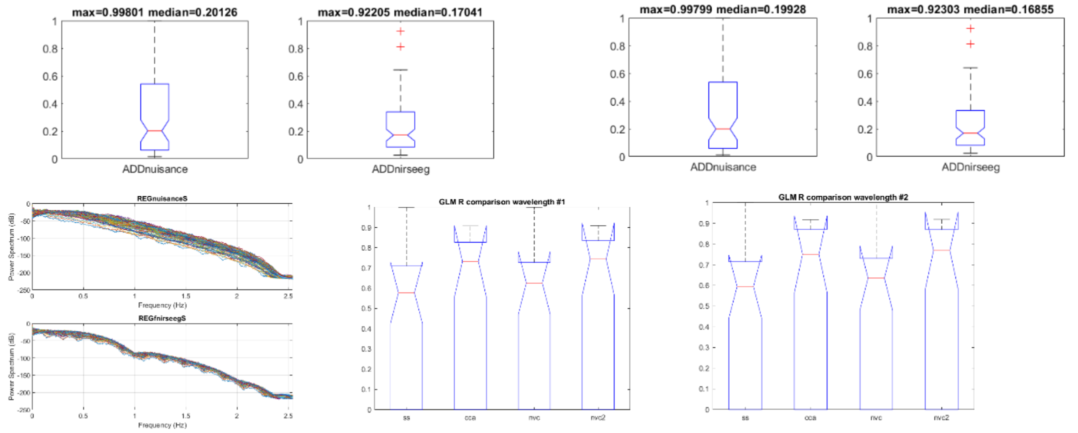

Figure S2: Illustrative example of the CER tDCS fNIRS-EEG dataset from the 12 subjects, (a)-(x), processed using temporally embedded CCA regularized with shrinkage of covariance matrices (‘rtcca’ function). In all the figures, (a)-(x), the top panel shows the ADDnuisance and ADDnirseeg, i.e., the CCA correlation coefficients between the projected fNIRS sources and the projected short separation fNIRS regressors and the EEG bandpower neurovascular coupling regressors respectively. The left two plots of the top panel show the box plot of the CCA correlation coefficients for param.tau=1 and the right two plots of the top panel show the box plot of the CCA correlation coefficients for param.tau=2. We selected param.tau=1 since the CCA correlation coefficients did not improve with param.tau=1. Other ‘rtcca’ parameter was param.NumOfEmb = ceil(timelag*Fs/param.tau) where timelag was the maximum timelag of 5sec for hemodynamic response and Fs was the sampling frequency of fNIRS data. The regressors, REGnuisanceS and REGfnirseegS, found using ‘rtcca’ function are shown in the left plots of the bottom panel. The top 5 regressors were used in the GLM design matrix to solve for the long separation fNIRS data where we compared GLM design matrix consisting of consecutive sequence of Gaussian as the basis function (width of the Gaussian and the step both equal to 0.5sec) along with solely short separation fNIRS channel data (labelled SS), solely REGnuisanceS data (labelled cca), solely REGfnirseegS data (labelled nvc), and REGnuisanceS with REGfnirseegS data (labelled nvc2) as regressors – see the middle and the right figures of the bottom panel corresponding to the first and the second wavelengths of the fNIRS data respectively. Illustrative subjects (P01) and conditions (POS vs. PRE) are (a) P01 POS, (b) P01 PRE.

Tests of Normality

	Stim region	Kolmogorov-Smirnov[a] Statistic	df	Sig.	Shapiro-Wilk Statistic	df	Sig.
TimeDiff	CER	.162	36	.018	.921	36	.014
	PFC	.129	36	.138	.960	36	.210
	SHM	.138	36	.082	.934	36	.032
DeviationDiff	CER	.178	36	.005	.958	36	.186
	PFC	.213	36	.000	.907	36	.005
	SHM	.244	36	.000	.796	36	.000
IncisionGapDiff	CER	.241	36	.000	.918	36	.011
	PFC	.265	36	.000	.880	36	.001
	SHM	.338	36	.000	.762	36	.000
KnotSecurityDiff	CER	.389	36	.000	.743	36	.000
	PFC	.428	36	.000	.662	36	.000
	SHM	.361	36	.000	.797	36	.000
ErrorDiff	CER	.148	36	.046	.925	36	.017
	PFC	.187	36	.003	.899	36	.003
	SHM	.234	36	.000	.920	36	.012
ScoreDiff	CER	.099	36	.200*	.964	36	.293
	PFC	.120	36	.200*	.962	36	.256
	SHM	.155	36	.029	.915	36	.009

*. This is a lower bound of the true significance.

a. Lilliefors Significance Correction

Figure S3: Test of Normality results from Kolmogorov-Smirnov and Shapiro-Wilk tests in IBM® SPSS® software platform.

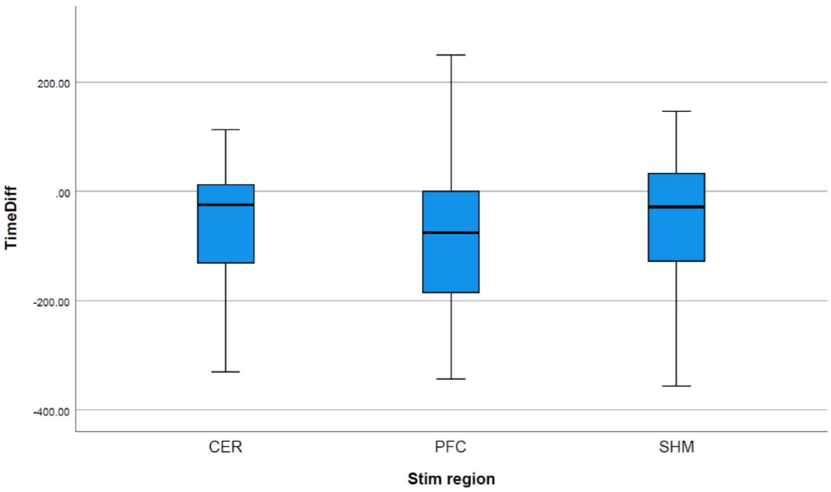
200.00
.00
-200.00
-400.00
TimeDiff
CER
PFC
SHM
Stim region

a)

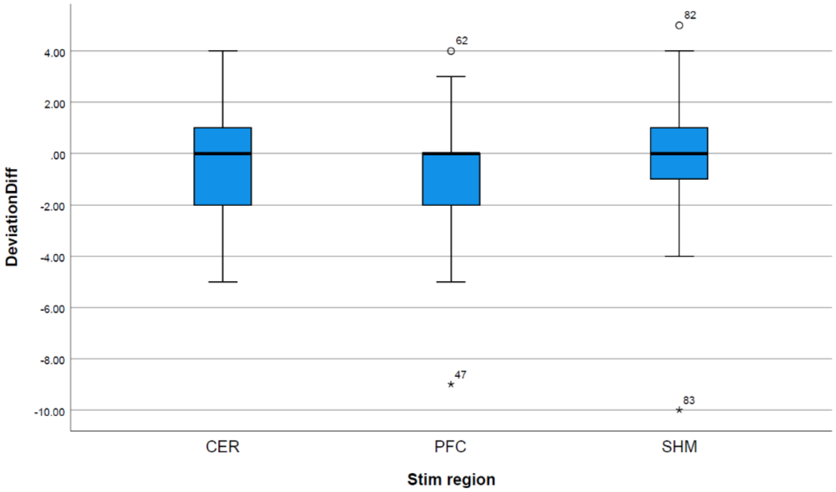
4.00
2.00
.00
-2.00
-4.00
-6.00
-8.00
-10.00
DeviationDiff
62
82
47
83
CER
PFC
SHM
Stim region

b)

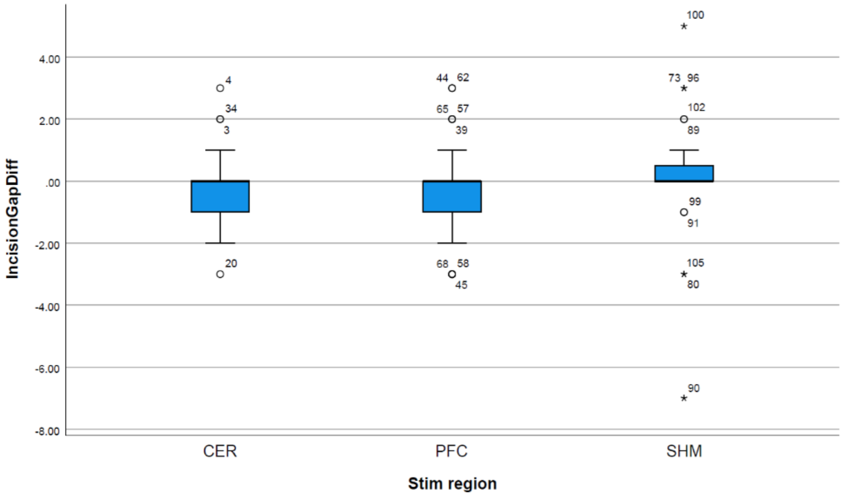
4.00
2.00
.00
-2.00
-4.00
-6.00
-8.00
IncisionGapDiff
4
34
3
20
44 62
65 57
39
68 58
45
100
73 96
102
89
99
91
105
80
90
CER
PFC
SHM
Stim region

c)

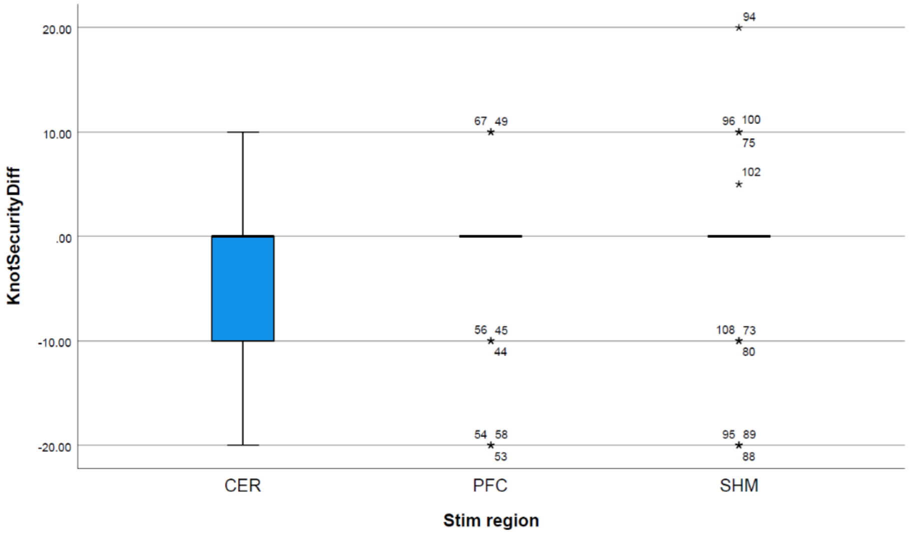

d)

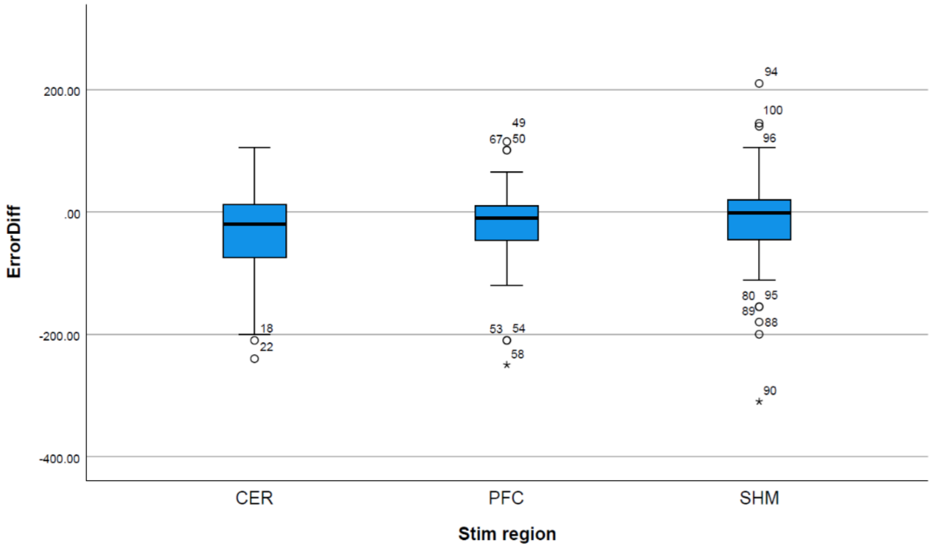

e)

Figure S4: The post-tDCS change in the FLS performance measures, namely, a) task time (TimeDiff), b) deviation (DeviationDiff), c) incision gap (IncisionGapDiff), d) knot security (KnotSecurityDiff), e) accuracy (ErrorDiff), from pre-tDCS baseline values.

	Stim region			Statistic	Std. Error
TimeDiff	CER	Mean		-69.1944	19.51311
		95% Confidence Interval for Mean	Lower Bound	-108.8082	
			Upper Bound	-29.5807	
		5% Trimmed Mean		-64.7284	
		Median		-25.0000	
		Variance		13707.418	
		Std. Deviation		117.07868	
		Minimum		-330.00	
		Maximum		113.00	
		Range		443.00	
		Interquartile Range		145.25	
		Skewness		-.749	.393
		Kurtosis		-.363	.768
	PFC	Mean		-82.7222	21.40418
		95% Confidence Interval for Mean	Lower Bound	-126.1750	
			Upper Bound	-39.2694	
		5% Trimmed Mean		-87.7099	
		Median		-75.5000	
		Variance		16493.006	
		Std. Deviation		128.42510	
		Minimum		-343.00	
		Maximum		250.00	
		Range		593.00	
		Interquartile Range		188.25	
		Skewness		.463	.393
		Kurtosis		.627	.768
	SHM	Mean		-58.7778	20.74169
		95% Confidence Interval for Mean	Lower Bound	-100.8856	
			Upper Bound	-16.6699	
		5% Trimmed Mean		-53.0185	
		Median		-28.5000	
		Variance		15487.835	
		Std. Deviation		124.45013	
		Minimum		-357.00	
		Maximum		146.00	

	Stim region			Statistic	Std. Error
		Range		503.00	
		Interquartile Range		166.00	
		Skewness		-.797	.393
		Kurtosis		-.007	.768
DeviationDiff	CER	Mean		-.5278	.36620
		95% Confidence Interval for Mean	Lower Bound	-1.2712	
			Upper Bound	.2157	
		5% Trimmed Mean		-.5062	
		Median		.0000	
		Variance		4.828	
		Std. Deviation		2.19722	
		Minimum		-5.00	
		Maximum		4.00	
		Range		9.00	
		Interquartile Range		3.00	
		Skewness		-.269	.393
		Kurtosis		-.227	.768
	PFC	Mean		-.7778	.39261
		95% Confidence Interval for Mean	Lower Bound	-1.5748	
			Upper Bound	.0193	
		5% Trimmed Mean		-.6605	
		Median		.0000	
		Variance		5.549	
		Std. Deviation		2.35568	
		Minimum		-9.00	
		Maximum		4.00	
		Range		13.00	
		Interquartile Range		2.00	
		Skewness		-1.101	.393
		Kurtosis		3.304	.768
	SHM	Mean		-.1111	.39797
		95% Confidence Interval for Mean	Lower Bound	-.9190	
			Upper Bound	.6968	
		5% Trimmed Mean		.0309	
		Median		.0000	

	Stim region			Statistic	Std. Error
		Variance		5.702	
		Std. Deviation		2.38780	
		Minimum		-10.00	
		Maximum		5.00	
		Range		15.00	
		Interquartile Range		2.00	
		Skewness		-1.701	.393
		Kurtosis		8.207	.768
IncisionGapDiff	CER	Mean		-.1111	.19832
		95% Confidence Interval for Mean	Lower Bound	-.5137	
			Upper Bound	.2915	
		5% Trimmed Mean		-.1235	
		Median		.0000	
		Variance		1.416	
		Std. Deviation		1.18990	
		Minimum		-3.00	
		Maximum		3.00	
		Range		6.00	
		Interquartile Range		1.00	
		Skewness		.118	.393
		Kurtosis		1.009	.768
	PFC	Mean		-.1667	.25973
		95% Confidence Interval for Mean	Lower Bound	-.6939	
			Upper Bound	.3606	
		5% Trimmed Mean		-.1852	
		Median		.0000	
		Variance		2.429	
		Std. Deviation		1.55839	
		Minimum		-3.00	
		Maximum		3.00	
		Range		6.00	
		Interquartile Range		1.00	
		Skewness		-.187	.393
		Kurtosis		.223	.768
	SHM	Mean		.1111	.31315

Stim region				Statistic	Std. Error
		95% Confidence Interval for Mean	Lower Bound	-.5246	
			Upper Bound	.7468	
		5% Trimmed Mean		.1852	
		Median		.0000	
		Variance		3.530	
		Std. Deviation		1.87887	
		Minimum		-7.00	
		Maximum		5.00	
		Range		12.00	
		Interquartile Range		.75	
		Skewness		-1.100	.393
		Kurtosis		6.213	.768
KnotSecurityDiff	CER	Mean		-3.0556	1.18318
		95% Confidence Interval for Mean	Lower Bound	-5.4575	
			Upper Bound	-.6536	
		5% Trimmed Mean		-2.8395	
		Median		.0000	
		Variance		50.397	
		Std. Deviation		7.09907	
		Minimum		-20.00	
		Maximum		10.00	
		Range		30.00	
		Interquartile Range		10.00	
		Skewness		-.998	.393
		Kurtosis		1.049	.768
	PFC	Mean		-1.6667	1.16155
		95% Confidence Interval for Mean	Lower Bound	-4.0247	
			Upper Bound	.6914	
		5% Trimmed Mean		-1.2963	
		Median		.0000	
		Variance		48.571	
		Std. Deviation		6.96932	
		Minimum		-20.00	
		Maximum		10.00	
		Range		30.00	

	Stim region			Statistic	Std. Error
		Interquartile Range		.00	
		Skewness		-1.370	.393
		Kurtosis		2.607	.768
	SHM	Mean		-1.8056	1.43859
		95% Confidence Interval for Mean	Lower Bound	-4.7261	
			Upper Bound	1.1149	
		5% Trimmed Mean		-1.7593	
		Median		.0000	
		Variance		74.504	
		Std. Deviation		8.63157	
		Minimum		-20.00	
		Maximum		20.00	
		Range		40.00	
		Interquartile Range		.00	
		Skewness		-.496	.393
		Kurtosis		1.266	.768
ErrorDiff	CER	Mean		-37.0278	12.80082
		95% Confidence Interval for Mean	Lower Bound	-63.0148	
			Upper Bound	-11.0407	
		5% Trimmed Mean		-33.8889	
		Median		-20.0000	
		Variance		5898.999	
		Std. Deviation		76.80494	
		Minimum		-240.00	
		Maximum		105.00	
		Range		345.00	
		Interquartile Range		93.00	
		Skewness		-.930	.393
		Kurtosis		.916	.768
	PFC	Mean		-25.8889	13.31164
		95% Confidence Interval for Mean	Lower Bound	-52.9129	
			Upper Bound	1.1352	
		5% Trimmed Mean		-21.9074	
		Median		-10.0000	
		Variance		6379.187	

Stim region				Statistic	Std. Error
		Std. Deviation		79.86981	
		Minimum		-250.00	
		Maximum		115.00	
		Range		365.00	
		Interquartile Range		58.00	
		Skewness		-1.025	.393
		Kurtosis		1.729	.768
	SHM	Mean		-16.8889	16.69744
		95% Confidence Interval for Mean	Lower Bound	-50.7865	
			Upper Bound	17.0087	
		5% Trimmed Mean		-14.3210	
		Median		-1.5000	
		Variance		10036.959	
		Std. Deviation		100.18462	
		Minimum		-310.00	
		Maximum		210.00	
		Range		520.00	
		Interquartile Range		82.75	
		Skewness		-.604	.393
		Kurtosis		1.565	.768

Figure S5: Descriptive statistics of post-tDCS change in the FLS performance measures, namely, task time (TimeDiff), deviation (DeviationDiff), incision gap (IncisionGapDiff), knot security (KnotSecurityDiff), accuracy (ErrorDiff), from pre-tDCS baseline values.

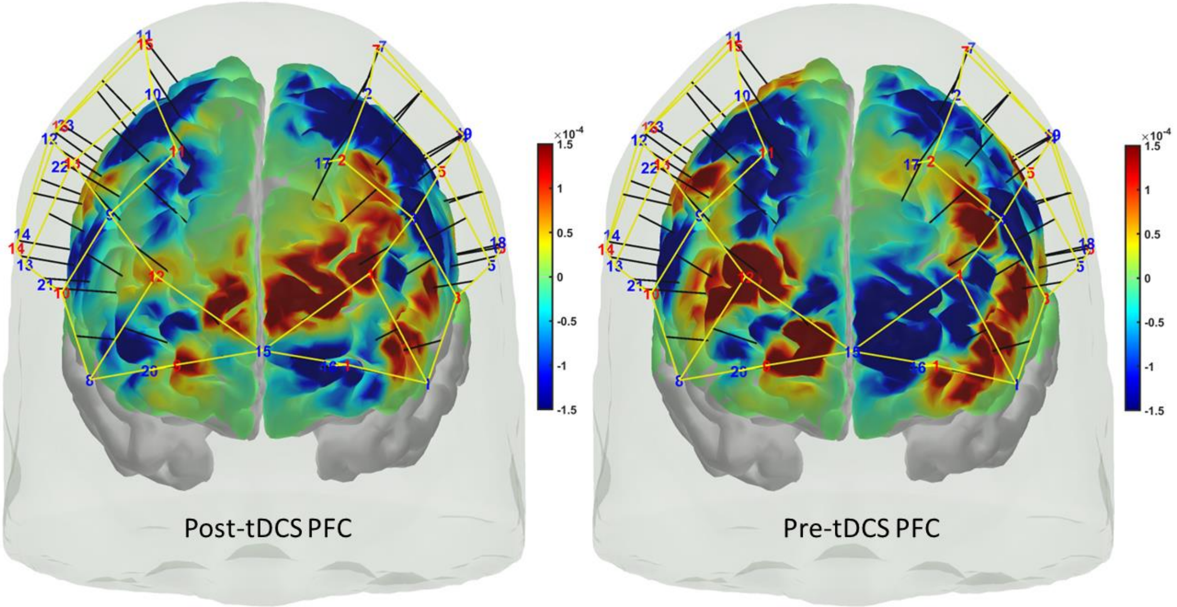

(a)

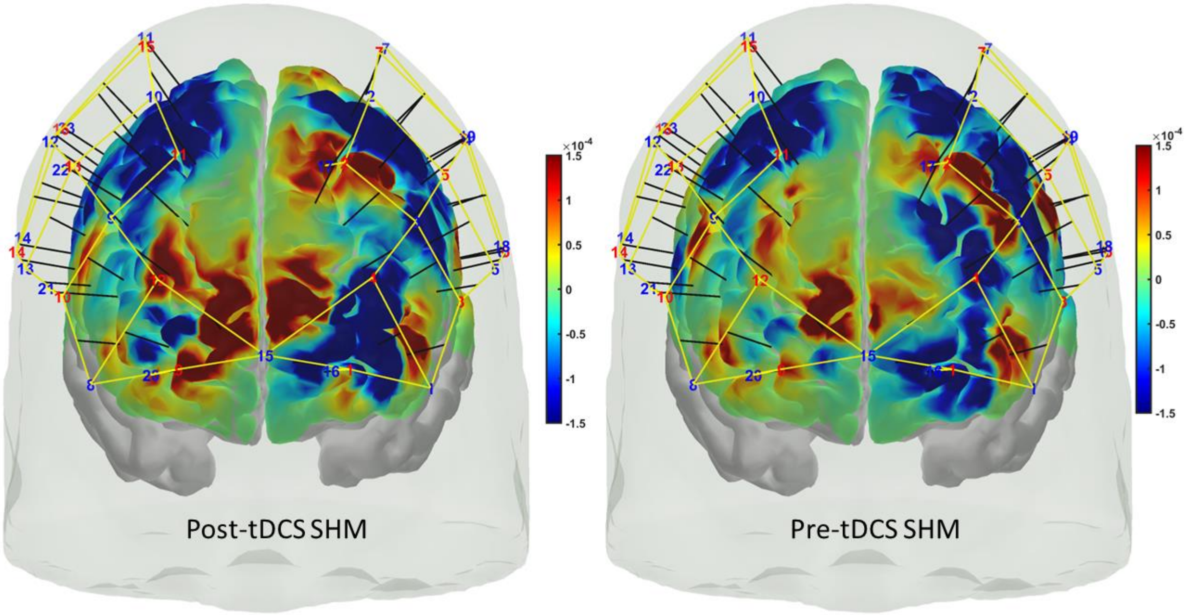

(b)

Figure S6: Cortical activation based on the oxyhemoglobin (HbO) hemodynamic response at the prefrontal areas at the start of the FLS task during post-tDCS (left panels) as well as pre-tDCS (right panels) baseline for (a) ventrolateral prefrontal cortex (PFC), (b) sham (SHM) tDCS.
